# Supplementary material for: A transcription-based mechanism for oncogenic β-catenin-induced lethality in BRCA1/2-deficient cells
Source: Nat Commun. 2021 Aug 13;12:4919. doi: 10.1038/s41467-021-25215-0 (PMC8363664; doi:10.1038/s41467-021-25215-0)
Supplement: Supplementary file 1 — Supplementary Information [file 41467_2021_25215_MOESM1_ESM.pdf]

Supplementary Information

A TRANSCRIPTION-BASED MECHANISM FOR ONCOGENIC β-CATENIN-INDUCED LETHALITY IN BRCA1/2-DEFICIENT CELLS

Dagg *et al.*

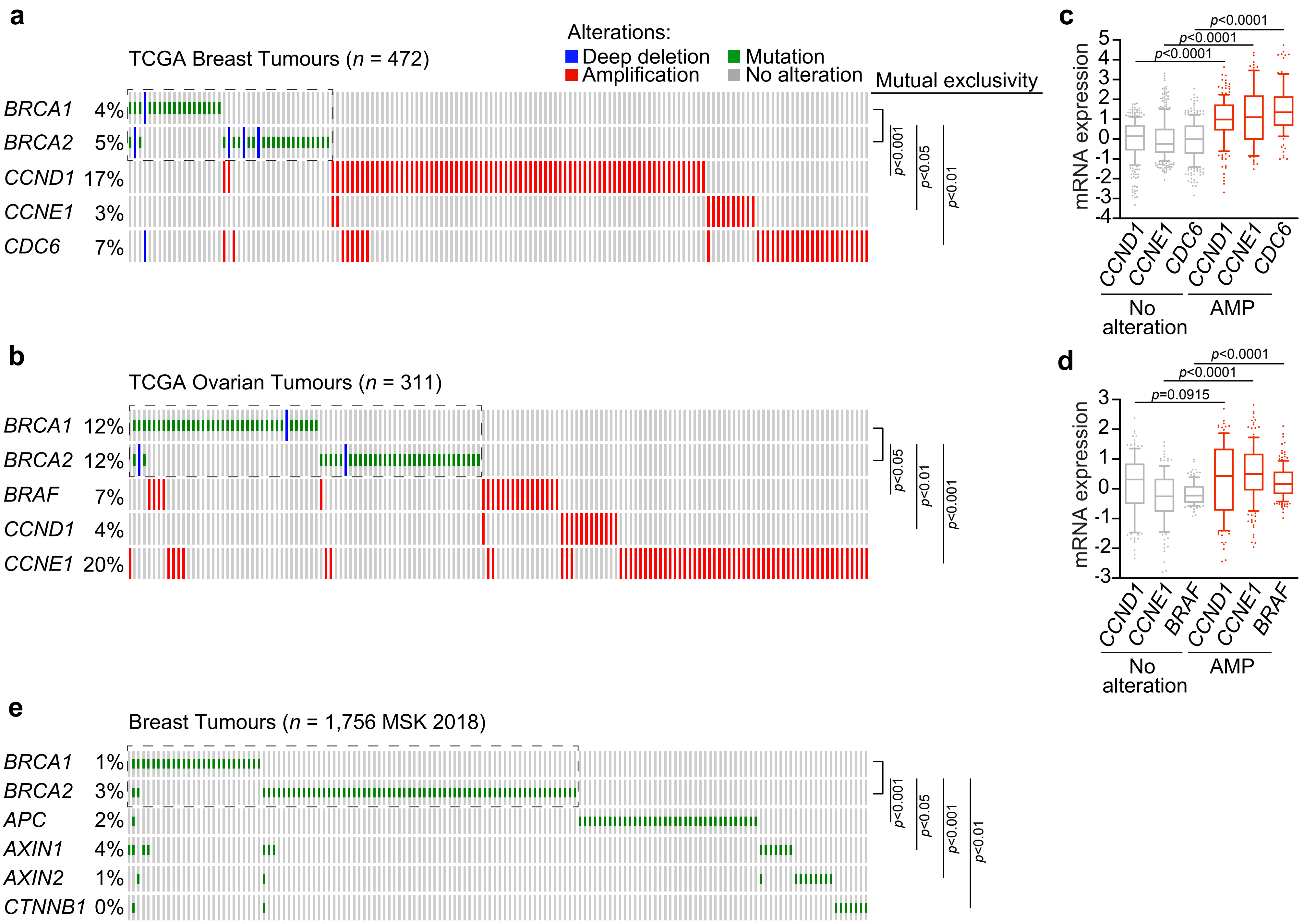
a
TCGA Breast Tumours (n = 472)
Alterations:
Deep deletion
Mutation
Amplification
No alteration
Mutual exclusivity
BRCA1 4%
BRCA2 5%
CCND1 17%
CCNE1 3%
CDC6 7%
p<0.001
p<0.05
p<0.01
c
mRNA expression
p<0.0001
p<0.0001
p<0.0001
CCND1
CCNE1
CDC6
CCND1
CCNE1
CDC6
No alteration
AMP
b
TCGA Ovarian Tumours (n = 311)
BRCA1 12%
BRCA2 12%
BRAF 7%
CCND1 4%
CCNE1 20%
p<0.05
p<0.01
p<0.001
d
mRNA expression
p=0.0915
p<0.0001
p<0.0001
CCND1
CCNE1
BRAF
CCND1
CCNE1
BRAF
No alteration
AMP
e
Breast Tumours (n = 1,756 MSK 2018)
BRCA1 1%
BRCA2 3%
APC 2%
AXIN1 4%
AXIN2 1%
CTNNB1 0%
p<0.001
p<0.05
p<0.001
p<0.01

Supplementary Fig. 1 Mutual exclusivity between BRCA1/2 mutations and oncogene or WNT pathway gene alterations in breast and ovarian tumours.

a,b, Alterations of the indicated oncogenes in the TCGA breast[1] (**a**) and TCGA ovarian tumour collections[2] (**b**). Only tumours with alterations in one of the indicated genes are shown. %, frequency of the indicated alterations in each gene. Red, amplification; blue, deletion; green, mutation (single-nucleotide variant); grey, no alteration. Statistical significance of mutual exclusivity between alterations in *BRCA1* and *BRCA2* genes, and amplifications of each oncogene were performed using the pairwise DISCOVER method. **c,d,** mRNA expression of indicated oncogenes (z-scores) in breast (**c**) or ovarian (**d**) tumours with no alterations or with amplifications (AMP) in the respective oncogenes. Boxes indicate the median value and 25th-75th percentile; whiskers indicate the 10th and 90th percentiles. Statistical significance was determined by a two-tailed Mann-Whitney test. **e**, Alterations of the indicated WNT pathway genes in the MSK breast tumour collection[3]. Only tumours with alterations in one of the indicated genes shown. %, frequency of the indicated alterations in each gene. Green, mutation (single-nucleotide variant); grey, no alteration. Mutual exclusivity tests between alterations in *BRCA1* and *BRCA2* genes, and in each of the WNT pathway genes were performed as in (**a** and **b**).

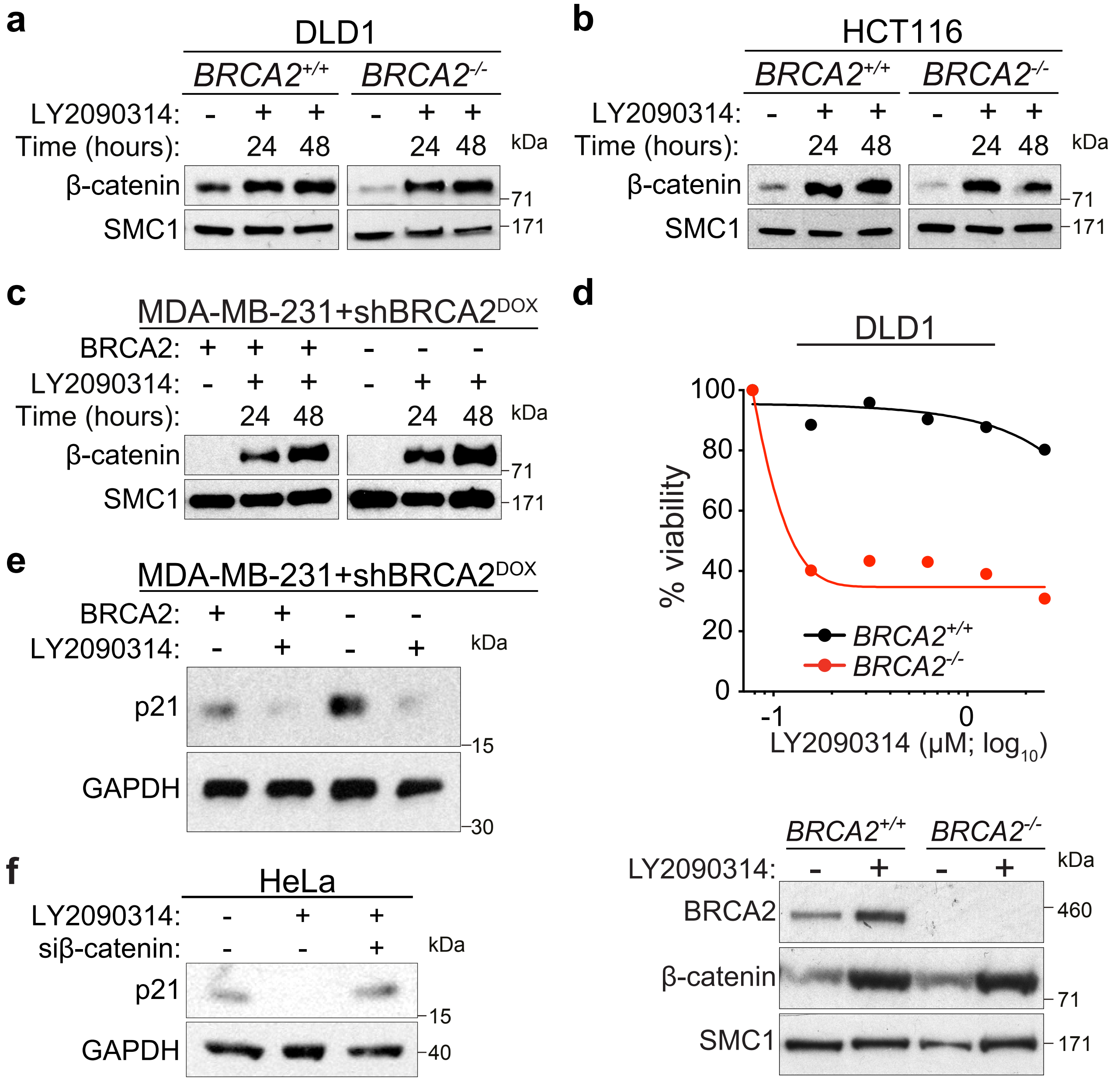

Supplementary Fig. 2 Effect of GSK3i on β-catenin and p21 levels in BRCA2-proficient and -deficient human cells.

a-c, BRCA2+/+ or BRCA2-/- human DLD1 cells (**a**) and HCT116 cells (**b**), as well as MDA-MB-231 cells carrying a DOX-inducible BRCA2 shRNA and grown in the presence (-BRCA2) or absence (+BRCA2) of DOX for 4 days (**c**) were incubated with 250 nM LY2090314. Whole-cell extracts prepared at indicated times after LY2090314 addition were immunoblotted as shown. SMC1 was used as a loading control. Data are representative of *n* = 2 independent experiments. **d**, BRCA2+/+ or BRCA2-/- human DLD1 cells were treated with LY2090314 at the indicated concentrations for 6 days and processed for cell viability assays. Whole-cell extracts prepared from cells treated with 250 nM LY2090314 were immunoblotted as shown. SMC1 was used as a loading control. Data are representative of *n* = 3 independent experiments, mean from one experiment plotted. **e**, MDA-MB-231 cells (+BRCA2 or -BRCA2) were treated with 2.5 μM LY2090314 for 48 hours and immunoblotted as shown. GAPDH was used as a loading control. Data are representative of *n* = 1 independent experiment. **f**, HeLa cells transfected with control or β-catenin siRNAs were grown in the presence or absence of 250 nM LY2090314 for 16 hours. Whole-cell extracts were immunoblotted as indicated. GAPDH was included as a loading control. Data are representative of *n* = 2 independent experiments. Source data for (**a-f**) are provided in the Source Data file.

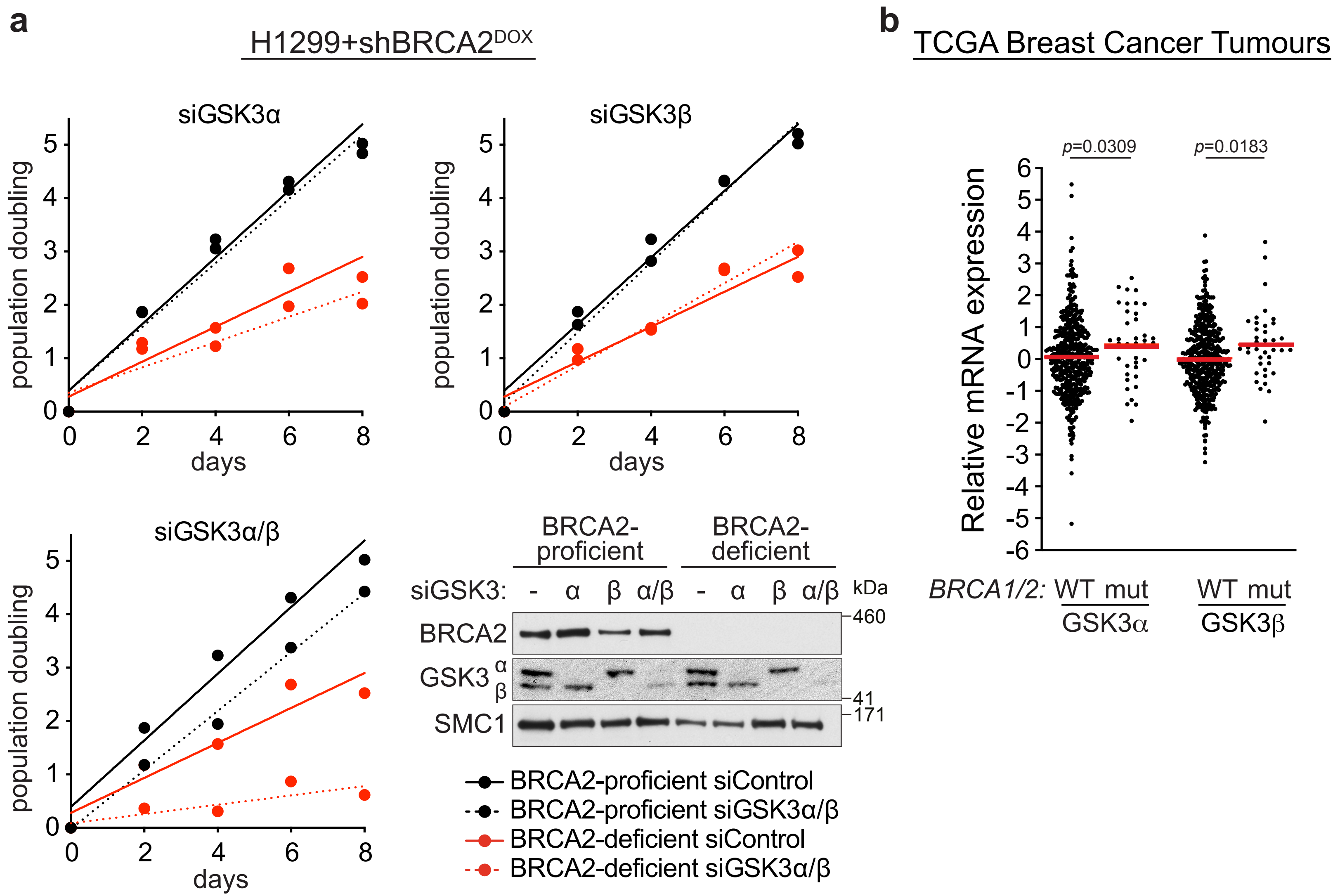

Supplementary Fig. 3 Effect of GSK3α/GSK3β depletion on BRCA2-deficient cell proliferation.
a, H1299 cells carrying DOX-inducible BRCA2 shRNA were transfected with control, GSK3α and/or GSK3β siRNAs and were processed for proliferation assays. Whole-cell extracts were immunoblotted as shown. SMC1 was used as a loading control. Data is representative of n = 3 independent experiments, n = 1 plotted in graph. **b**, GSK3α and GSK3β mRNA expression in the TCGA breast tumour collection (n = 443 for WT; n = 38 for BRCA1/2-mutated[1]). Each dot represents a single tumour. Middle line, mean. Statistical significance was determined by a two-tailed Mann-Whitney test. Source data for (**a**) is provided in the Source Data file.

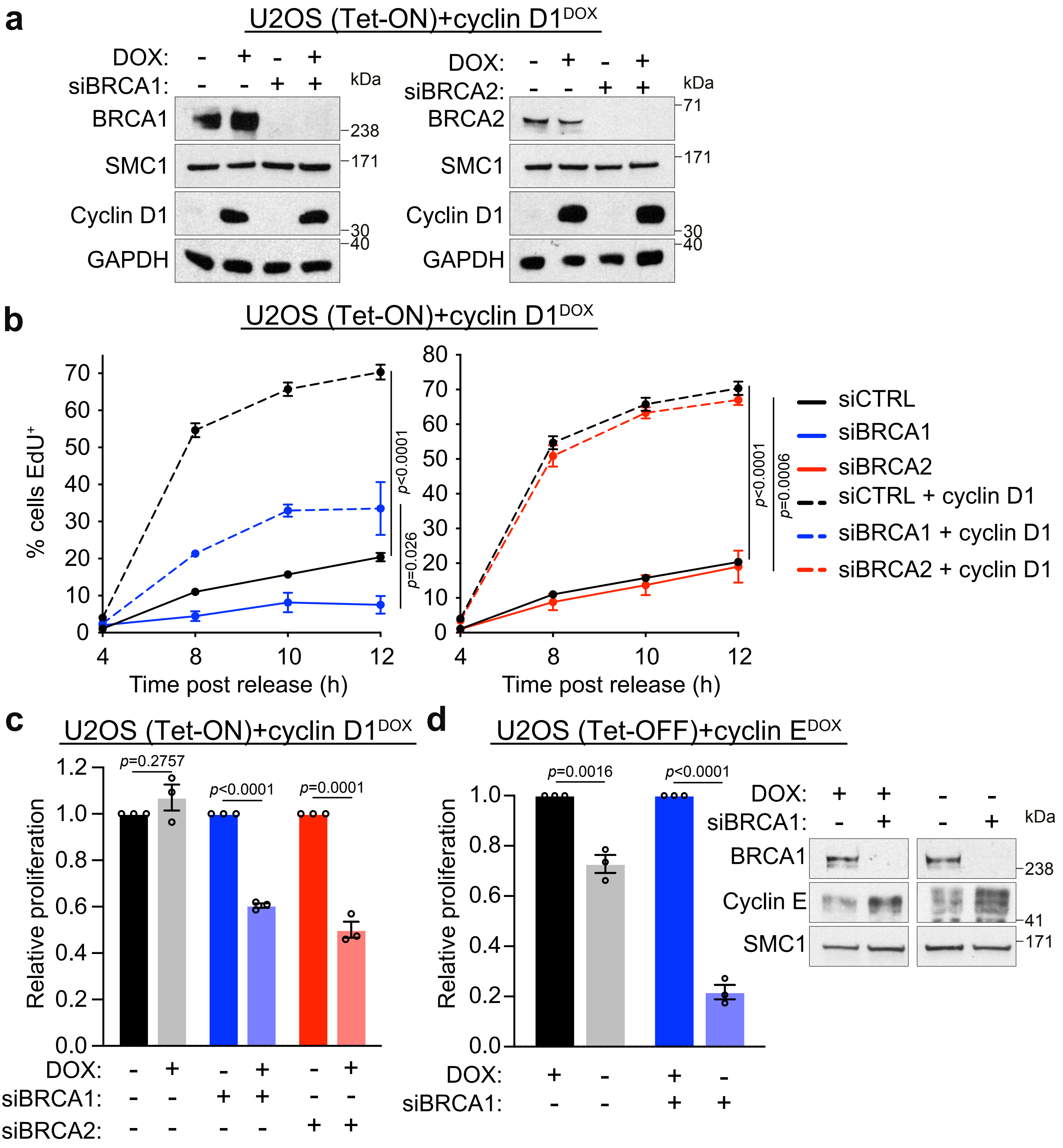

Supplementary Fig. 4 Oncogenic cyclin D1 and cyclin E overexpression decrease survival in BRCA1/2 -deficient cells.

a, Human U2OS cells carrying a tetracycline-inducible (Tet-ON) cassette controlling cyclin D1 expression were cultured in the presence (cyclin D1 overexpression) or absence (endogenous cyclin D1 expression) of 2 μg/mL DOX. Cells were transfected with control, BRCA1 or BRCA2 siRNAs for two days prior to whole-cell extract preparation. Extracts were immunoblotted as shown. SMC1 and GAPDH were used as loading controls. Data are representative of n = 3 independent experiments. **b**, Cells treated as in (**a**) were arrested in mitosis with nocodazole treatment. Mitotic cells were collected and released in fresh medium containing 25 μM EdU. The % of EdU-positive cells was determined using FACS analyses at the indicated time points after mitotic shake-off. Error bars represent SEM of n = 3 independent experiments. Statistical significance was determined by an unpaired two-tailed t-test. **c**, Proliferation assays were performed in cells treated as in (**a**). Error bars represent SEM of n = 3 independent experiments. Statistical significance was determined by an unpaired two-tailed t-test. **d**, Human U2OS cells with an inducible cyclin E expression (tetracycline (TET)-OFF system) were grown in the presence or absence of TET. Cells were transfected with control or BRCA1 siRNAs for two days prior to seeding for proliferation assays. Whole-cell extracts were prepared at the same time as seeding and immunoblotted as indicated. SMC1 was used as a loading control. Error bars represent SEM of n = 3 independent experiments. Statistical significance was determined by an unpaired two-tailed t-test. Source data for (**a-d**) is provided in the Source Data file.

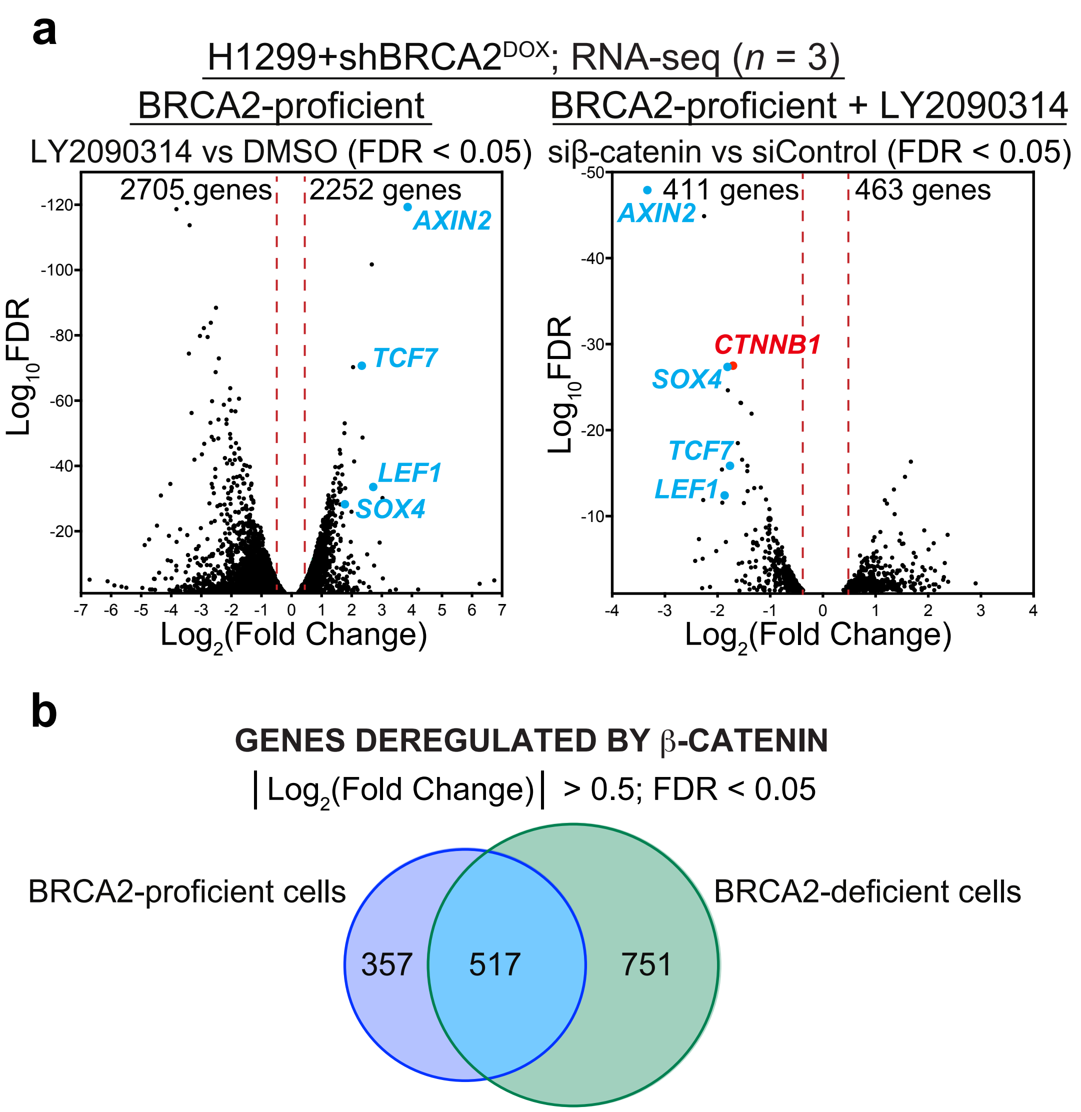

Supplementary Fig. 5 Oncogenic β-catenin deregulates transcription of WNT pathway gene targets in BRCA2-proficient cells.

a, BRCA2-proficient H1299 cells transfected with control or β-catenin siRNAs were treated with DMSO or LY2090314 for 24 hours before processing for RNA-seq. *n* = 3 independent experiments. Volcano plots show genes differentially expressed (FDR < 0.05) in the indicated samples. Gene subsets significantly downregulated (log2(Fold Change) < −0.5) or upregulated (log2(Fold Change) > 0.5) are marked by dotted lines. **b**, Venn diagram of genes significantly upregulated or downregulated by LY2090314 and β-catenin siRNAs (FDR < 0.05 and |log2(Fold Change)| > |0.5|) in H1299 BRCA2-proficient (purple) and -deficient (green) cells. Genes overlapping in the two samples of Venn diagram are indicated in blue (517 genes).

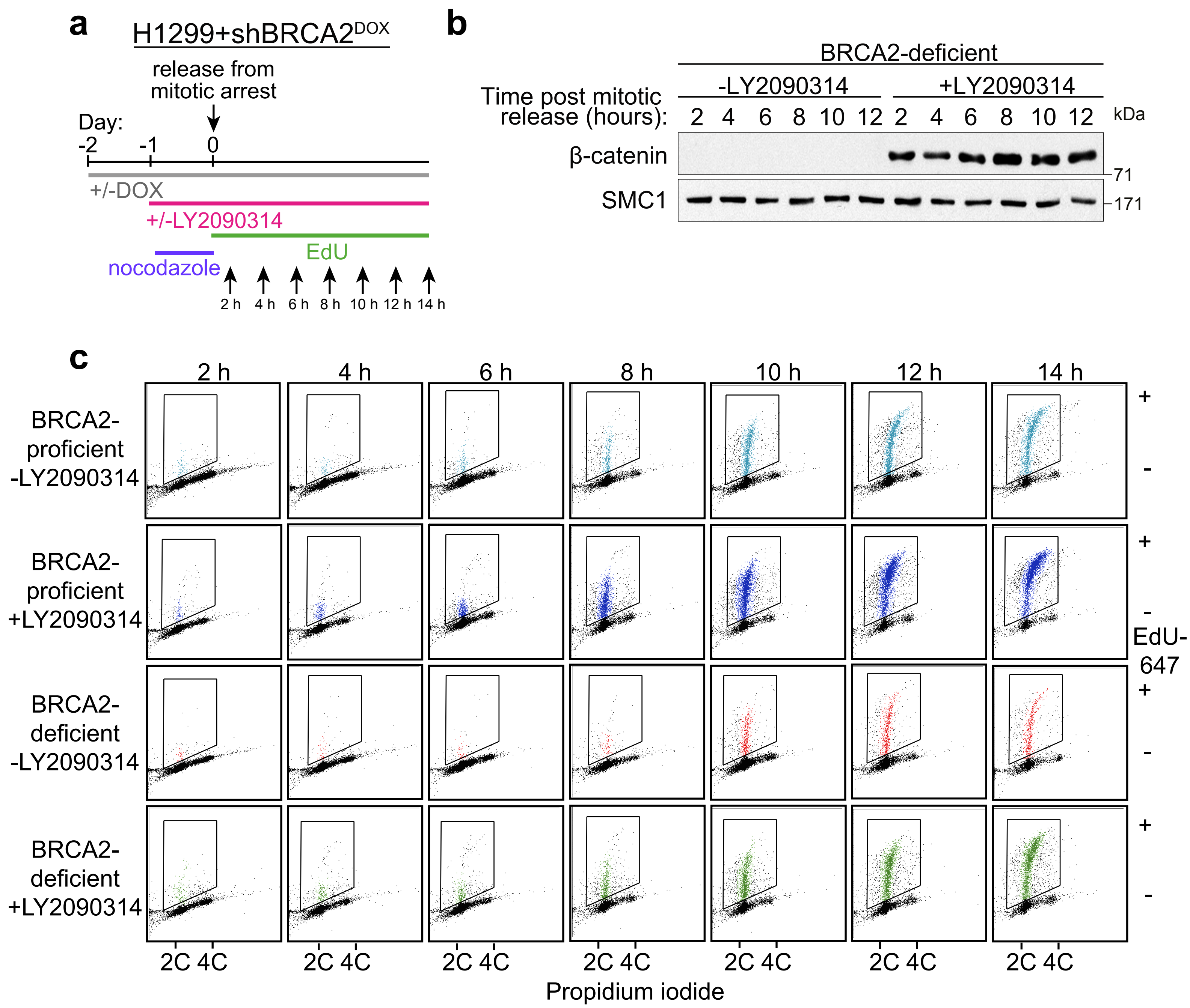

Supplementary Fig. 6 β-catenin activation accelerates the G1/S transition in BRCA2-proficient and -deficient H1299 cells.

a, Diagram of S-phase entry experimental conditions. H1299 cells expressing a DOX-inducible BRCA2 shRNA were grown in the presence or absence of LY2090314 for 24 hours and 100 ng/mL nocodazole was added during the final 8 hours of treatment. Mitotic cells were collected by mitotic shake-off and released in fresh medium containing 25 µM EdU, in the presence or absence of LY2090314. The % of EdU-positive cells was determined using FACS analyses at the indicated time points after mitotic shake-off. **b**, Whole-cell extracts prepared from BRCA2-deficient H1299 cells at the indicated time points after release from mitotic arrest were immunoblotted as shown. SMC1 was used as a loading control. Data are representative of *n* = 2 independent experiments. **c**, Example flow cytometry plots of DNA content (detected by propidium iodide) vs EdU incorporation (detected by Alexa Fluor 647) in BRCA2-proficient and -deficient H1299 cells, grown in the presence or absence of LY2090314 and prepared at the indicated time points after release from mitotic arrest. Boxes indicate EdU+ cells in each sample, figure exemplifies gating strategy used to determine the % EdU + cells. 2C and 4C indicate the DNA content of G1 and G2 cells, respectively. Source data for (**b**) is provided in the Source Data file.

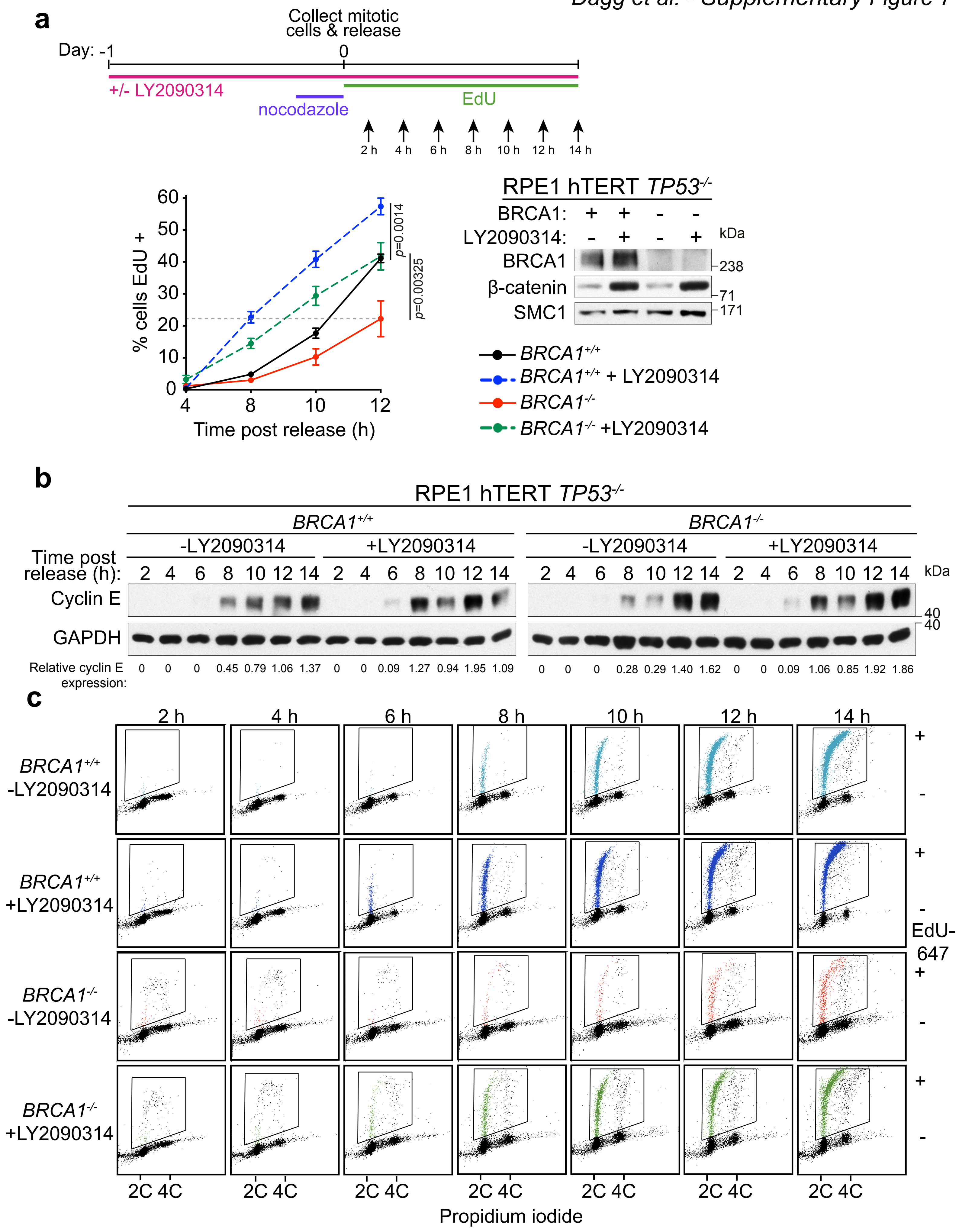
a
Collect mitotic cells & release
Day: -1
0
+/- LY2090314
nocodazole
EdU
2 h
4 h
6 h
8 h
10 h
12 h
14 h
% cells EdU +
Time post release (h)
p=0.0014
p=0.00325
RPE1 hTERT TP53-/-
BRCA1: + + - -
LY2090314: - + - +
kDa
BRCA1
β-catenin
SMC1
238
71
171
BRCA1+/+
BRCA1+/+ + LY2090314
BRCA1-/-
BRCA1-/- +LY2090314
b
RPE1 hTERT TP53-/-
BRCA1+/+
BRCA1-/-
-LY2090314
+LY2090314
Time post release (h): 2 4 6 8 10 12 14 2 4 6 8 10 12 14 2 4 6 8 10 12 14 2 4 6 8 10 12 14
kDa
Cyclin E
GAPDH
40
40
Relative cyclin E expression: 0 0 0 0.45 0.79 1.06 1.37 0 0 0.09 1.27 0.94 1.95 1.09 0 0 0 0.28 0.29 1.40 1.62 0 0 0.09 1.06 0.85 1.92 1.86
c
2 h
4 h
6 h
8 h
10 h
12 h
14 h
BRCA1+/+ -LY2090314
BRCA1+/+ +LY2090314
BRCA1-/- -LY2090314
BRCA1-/- +LY2090314
EdU-647
2C 4C
Propidium iodide

Supplementary Fig. 7 β-catenin activation accelerates the G1/S transition in *BRCA1*$^{+/+}$ and *BRCA1*$^{-/-}$ RPE1 cells.

a, *BRCA1*$^{+/+}$ or *BRCA1*$^{-/-}$ human RPE1 cells, hTERT-immortalised and TP53-deleted, were grown in the presence or absence of 100 nM LY2090314 for 24 hours and 100 ng/mL nocodazole was added in the final 8 hours of treatment. Mitotic cells were collected and released in fresh medium containing 25 µM EdU in the presence or absence of 100 nM LY2090314. The % of EdU-positive cells was determined using FACS analysis at the indicated time points after mitotic shake-off. Error bars represent SEM of *n* = 4 independent experiments. Statistical significance was determined by an unpaired two-tailed *t*-test). Whole-cell extracts prepared at the time of mitotic shake-off were immunoblotted as shown. SMC1 was used as a loading control. **b**, Whole-cell extracts prepared from BRCA1-proficient or -deficient RPE1 cells at indicated time points after release from mitotic arrest were immunoblotted as indicated. GAPDH was used as a loading control. Cyclin E expression was quantified relative to GAPDH control. Data are representative of *n* = 2 independent experiments. **c**, Example flow cytometry plots of DNA content (detected by propidium iodide) vs EdU incorporation (detected by Alexa Fluor 647) in BRCA1-proficient and -deficient RPE1 cells, grown in the presence or absence of 100 nM LY2090314 and prepared at the indicated time points after release from mitotic arrest. Boxes indicate EdU+ cells in each sample, figure exemplifies gating strategy used to determine the % EdU + cells. 2C and 4C indicate the DNA content of G1 and G2 cells, respectively. Source data for (**a-b**) is provided in the Source Data file.

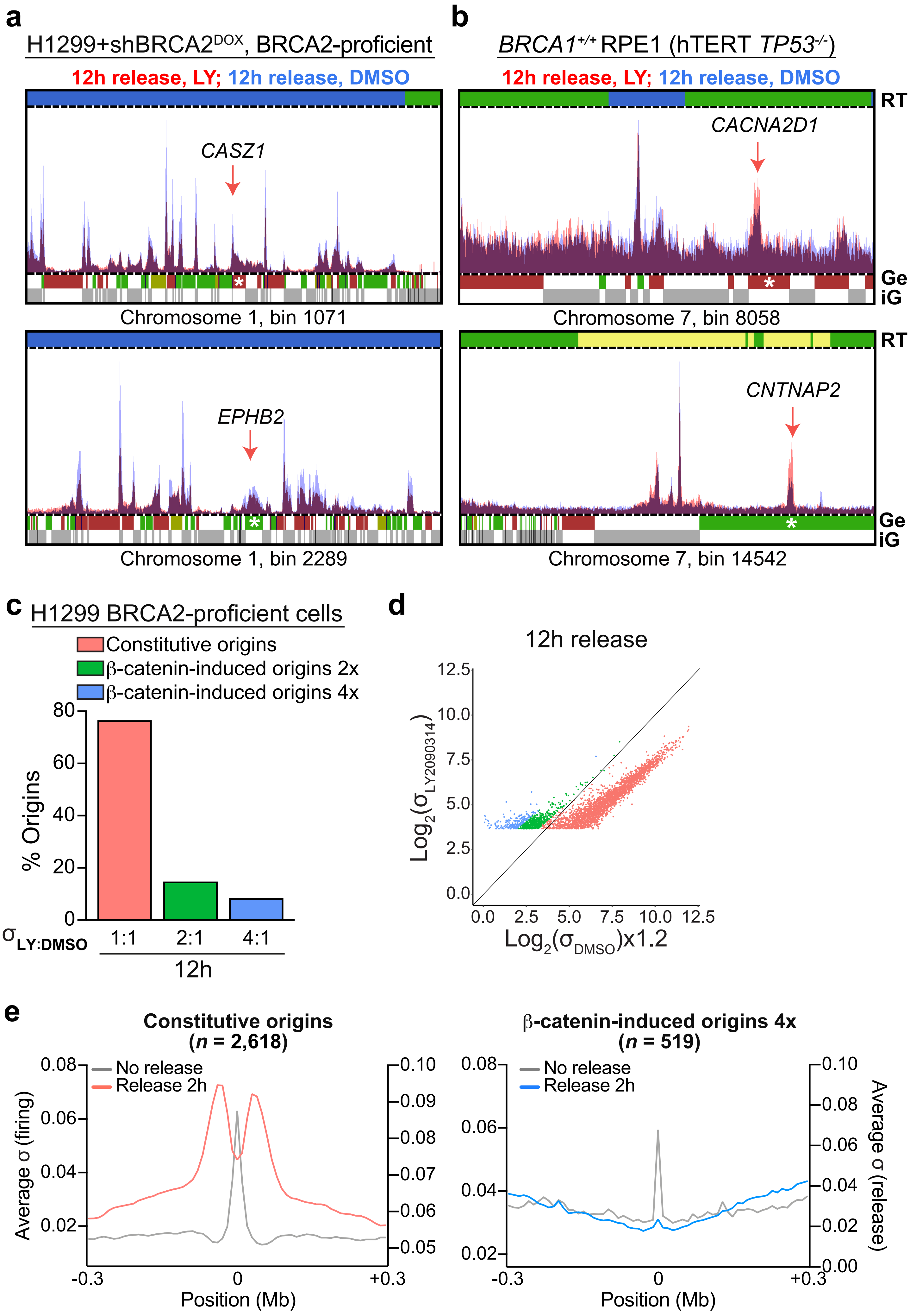
a
H1299+shBRCA2DOX, BRCA2-proficient
12h release, LY; 12h release, DMSO
CASZ1
Chromosome 1, bin 1071
EPHB2
Chromosome 1, bin 2289
b
BRCA1+/+ RPE1 (hTERT TP53-/-)
12h release, LY; 12h release, DMSO
RT
CACNA2D1
Ge
iG
Chromosome 7, bin 8058
RT
CNTNAP2
Ge
iG
Chromosome 7, bin 14542
c
H1299 BRCA2-proficient cells
Constitutive origins
β-catenin-induced origins 2x
β-catenin-induced origins 4x
% Origins
80
60
40
20
0
σLY:DMSO
1:1
2:1
4:1
12h
d
12h release
Log2(σLY2090314)
12.5
10.0
7.5
5.0
2.5
0.0
Log2(σDMSO)x1.2
e
Constitutive origins
(n = 2,618)
No release
Release 2h
Average σ (firing)
0.08
0.06
0.04
0.02
0.10
0.09
0.08
0.07
0.06
0.05
-0.3
0
+0.3
Position (Mb)
β-catenin-induced origins 4x
(n = 519)
No release
Release 2h
0.08
0.06
0.04
0.02
0.10
0.08
0.06
0.04
0.02
0.00
Average σ (release)
-0.3
0
+0.3
Position (Mb)

Supplementary Fig. 8 β-catenin activation triggers aberrant origin firing and fork collapse in BRCA1/2-proficient human cells.
a,**b**, BRCA2-proficient H1299 (**a**) and *BRCA1*$^{+/+}$ RPE1 (**b**) cells were grown in the presence (red) or absence (blue) of LY2090314 for 24 hours and 100 ng/mL nocodazole was added during the final 8 hours of treatment. Mitotic cells were collected and released in fresh medium containing 25 μM EdU and 2 mM HU. EdU-labelled DNA was isolated from cells at 12 hours after mitotic release and analysed using high-throughput sequencing. RT, replication timing: blue, early; green, mid; yellow, late S-phase. Ge, genes (green, forward direction of transcription; purple, reverse direction of transcription); iG, intergenic regions (grey). β-catenin-induced origins firing within genes (indicated with white stars) in BRCA1/2-deficient cells (Fig. 4a) are shown by red arrows. **c**, Origin classification based on adjusted σ value ratios in BRCA2-proficient H1299 cells released from mitotic arrest for 12 hours. **d**, Scatter plots of EdU-seq σ values at 12 hours after BRCA2-deficient H1299 cells release from mitotic arrest, color-coded as in (**c**). **e**, Genome-wide average fork progression in BRCA2-proficient H1299 cells treated with hydroxyurea for 12 hours after mitotic shake-off and released for 2 hours. EdU was added 30 min before cell collection.

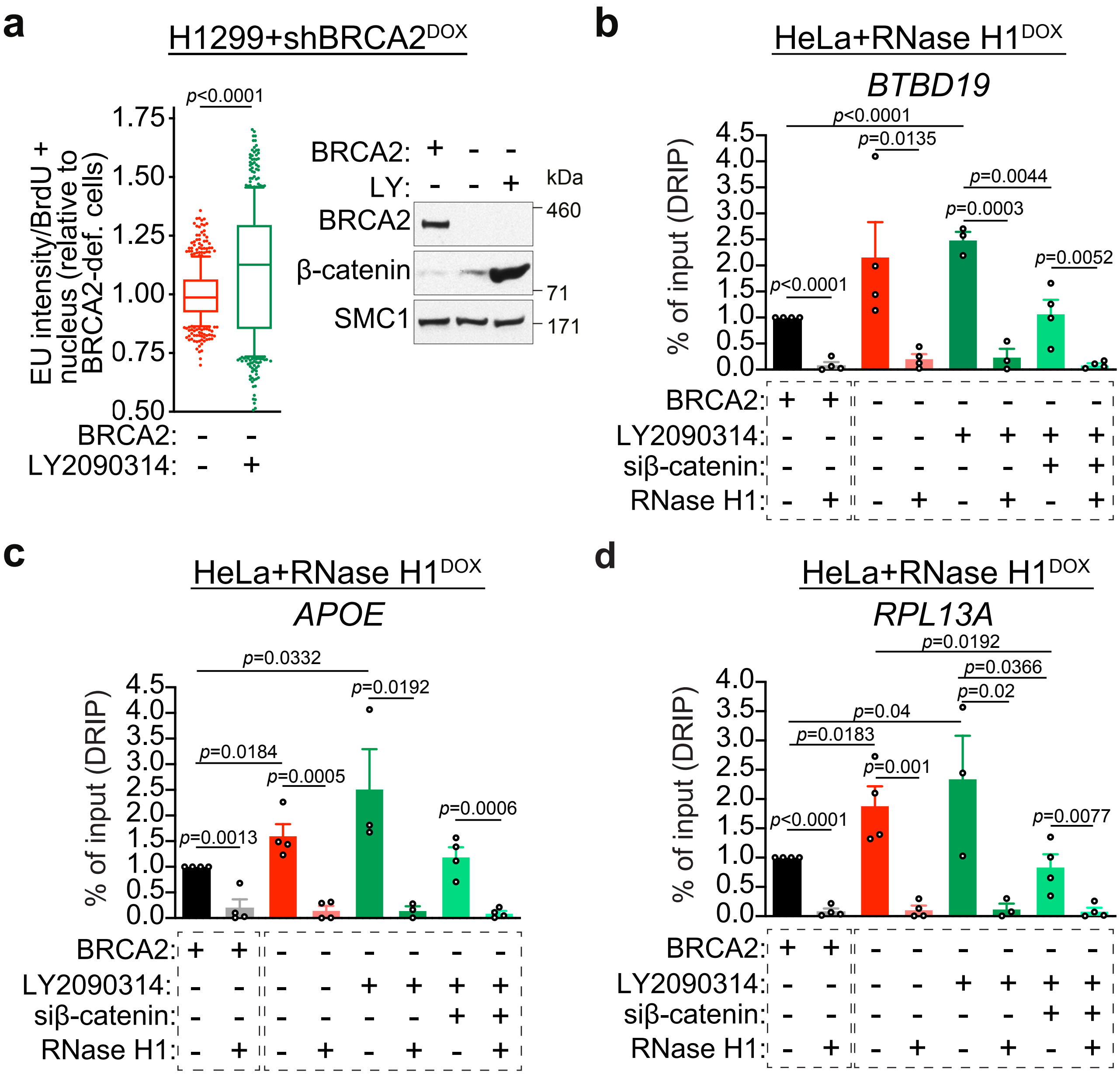

Supplementary Fig. 9 β-catenin-dependent increase in S-phase transcription in BRCA2-deficient cells and in DNA-RNA hybrids (R-loops) at specific genomic loci.
a, Human H1299 cells carrying a doxycycline (DOX)-inducible BRCA2 shRNA grown in the presence (-BRCA2) or absence (+BRCA2) of 2 µg/mL DOX and 250 nM LY2090314 were pulsed with 1 mM 5-ethynyl uridine (EU) and 1 µM BrdU for 1 h. EU incorporation in BrdU positive cells (≥500 cells analysed in one experiment) was quantified as immunofluorescence intensity. Statistical significance was determined by a two-tailed Mann-Whitney test. Boxes indicate the median value and 25th-75th percentile; whiskers indicate the 10th and 90th percentiles. Whole-cell extracts prepared from these samples were immunoblotted as indicated. SMC1 was included as a loading control. **b-d**, HeLa cells carrying a DOX-inducible FLAG-tagged RNaseH1 were grown in the presence (+RNaseH1) or absence (-RNaseH1) of DOX and transfected with control, BRCA2 or β-catenin siRNAs. Cells were treated with LY2090314 for 16 h followed by DNA:RNA immunoprecipitation (DRIP)-qPCR using the anti S9.6 antibody at *BTBD19* (**b**), *APOE* (**c**) and *RPL13A* (**d**) loci. Error bars represent SEM of n = 4 independent experiments. Statistical significance was determined by an unpaired one-tailed t-test. Source data for (**a-d**) is provided in the Source Data file.

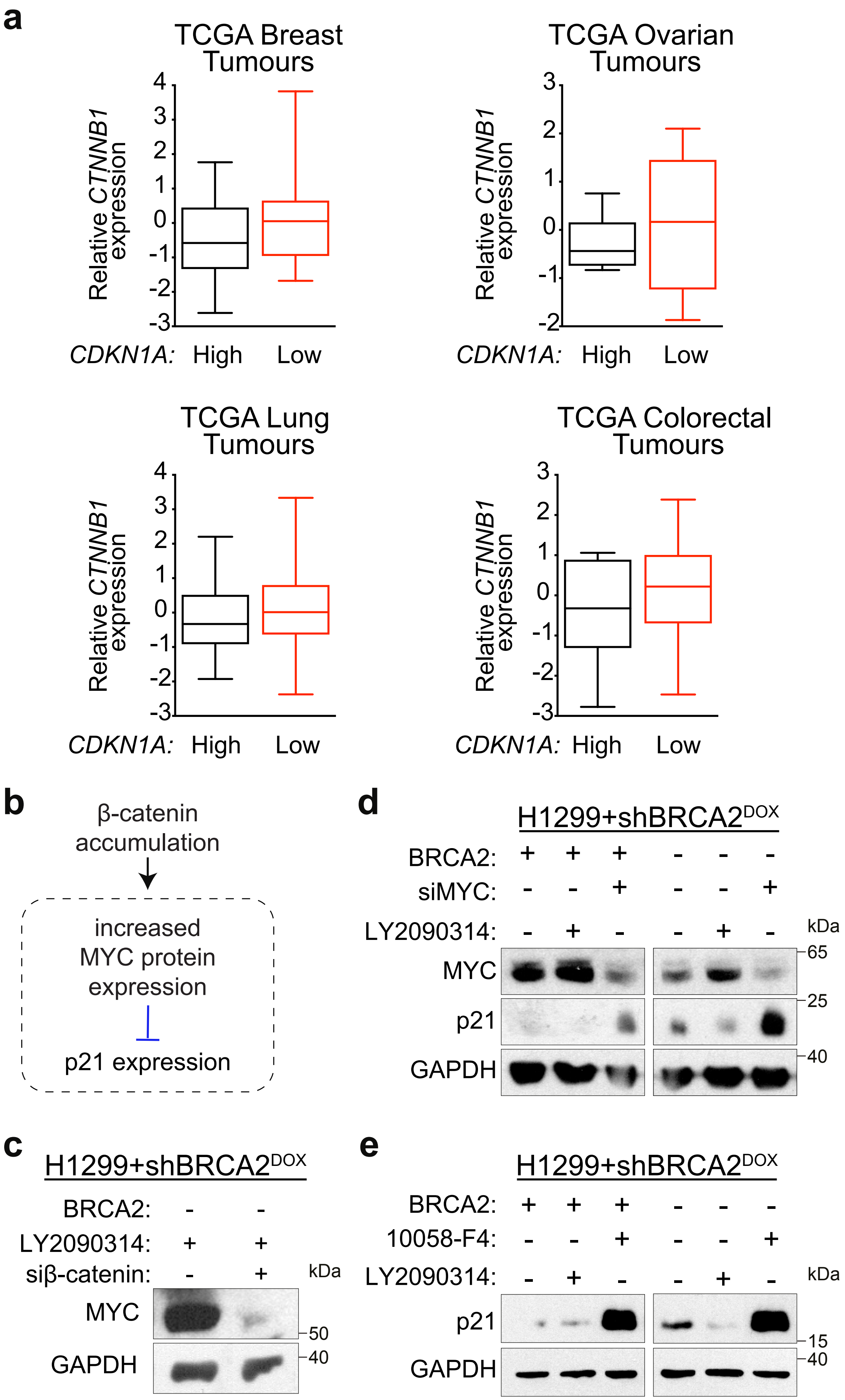
a
TCGA Breast Tumours
TCGA Ovarian Tumours
TCGA Lung Tumours
TCGA Colorectal Tumours
Relative CTNNB1 expression
CDKN1A: High Low
b
β-catenin accumulation
increased MYC protein expression
p21 expression
c
H1299+shBRCA2DOX
BRCA2: - -
LY2090314: + +
siβ-catenin: - +
MYC
GAPDH
kDa
50
40
d
H1299+shBRCA2DOX
BRCA2: + + + - - -
siMYC: - - + - - +
LY2090314: - + - - + -
MYC
p21
GAPDH
kDa
65
25
40
e
H1299+shBRCA2DOX
BRCA2: + + + - - -
10058-F4: - - + - - +
LY2090314: - + - - + -
p21
GAPDH
kDa
15
40

Supplementary Fig. 10 Correlation between *CTNNB1* and *CDKN1A* expression in TCGA tumours and MYC impact on p21 expression.

a, *CTNNB1* mRNA expression in tumour subsets with high or low CDKN1A expression from breast (*n* = 12 for high; *n* = 11 for low;[1]), ovarian (*n* = 12 for high; *n* = 6 for low;[2]), lung (*n* = 29 for high; *n* = 34 for low; PanCancer Atlas) and colorectal (*n* = 13 for high; *n* = 14 for low; PanCancer Atlas) TCGA tumour collections. Boxes indicate the median value and 25th-75th percentile. Whiskers indicate the 10th and 90th percentiles. **b**, Diagrammatic representation of MYC-dependent downregulation of p21 expression, and of MYC activation through β-catenin accumulation. **c**, Human H1299 cells carrying a doxycycline (DOX)-inducible BRCA2 shRNA and grown in the presence of 2 µg/mL DOX (-BRCA2) and 250 nM LY2090314 were treated with control or β-catenin siRNAs. Whole-cell extracts prepared 24 hours after LY2090314 addition were immunoblotted as shown. GAPDH was used as a loading control. Data are representative of *n* = 2 independent experiments. **d**, Human H1299 cells carrying a doxycycline (DOX)-inducible BRCA2 shRNA were grown in the presence (-BRCA2) or absence (+BRCA2) of 2 µg/mL DOX and transfected with control or MYC siRNA. Whole cells extracts prepared after 24 hours treatment with 250 nM LY2090314 were immunoblotted as indicated. GAPDH was used as a loading control. Data are representative of *n* = 2 independent experiments. **e**, Human H1299 cells carrying a doxycycline (DOX)-inducible BRCA2 shRNA and grown in the presence (-BRCA2) or absence (+BRCA2) of 2 µg/mL DOX were treated with 250 nM LY2090314 and 10 µM MYC inhibitor 10058-F4 as indicated. Whole cell extracts prepared after 24 hours treatment were immunoblotted as shown. GAPDH was used as a loading control. Data from *n* = 1 independent experiment. Source data for (**c-e**) is provided in the Source Data file.

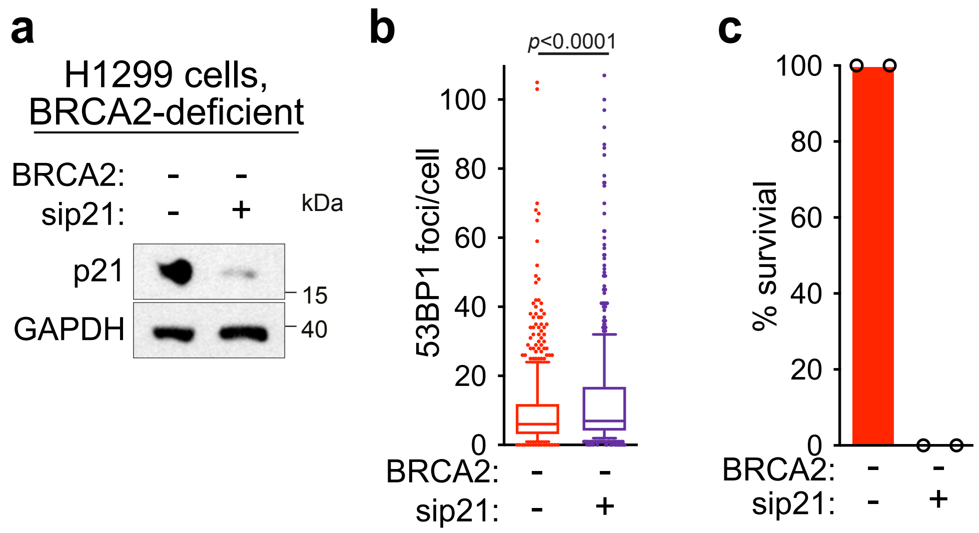

Supplementary Fig. 11 Downregulation of p21 expression with a second siRNA (Ambion) induces DNA damage and impairs the survival of BRCA2-deficient cells. **a**, Human H1299 cells carrying a doxycycline (DOX)-inducible BRCA2 shRNA and grown in the presence of 2 µg/mL DOX (-BRCA2) were transfected twice with control siRNA or p21 siRNA (Ambion). One day after the second transfection whole-cell extracts were prepared and immunoblotted as shown. GAPDH was used as a loading control. Data are representative of n = 3 independent experiments. **b**, The number of 53BP1 foci per cell were quantified in cells treated as in (**a**) after a further two days of growth. Data were obtained from n = 3 independent experiments. Two hundred cells were quantified per experiment. Boxes indicate the median value and 25th-75th percentile. Whiskers indicate the 10th and 90th percentiles. Statistical significance was determined by a two-tailed Mann-Whitney test. **c**, Clonogenic survival assays performed in the cells described in (**a**). n = 2 independent experiments. Source data for (**a-c**) is provided in the Source Data file.

Supplementary Table 1. PCR primers.

**Primer Name**	**Sequence**
CDKN1A forward	CAC TGT CTT GTA CCC TTG TGC
CDKN1A reverse	TTA GGG CTT CCT CTT GGA GA
GAPDH forward	GAC AGT CAG CCG CAT CTT CT
GAPDH reverse	ACC AAA TCC GTT GAC TCC GA
BTBD19 forward	CCC CAA AGG GTG GTG ACT T
BTBD19 reverse	TTC ACA TTA CCC AGA CCA GAC TGT
APOE forward	GGG AGC CCT ATA ATT GGA CAA GT
APOE reverse	CCC GAC TGC GCT TCT CA
RPL13A forward	GCT TCC AGC ACA GGA CAG GTA T
RPL13A reverse	CAC CCA CTA CCC GAG TTC AAG

Supplementary References

1 Koboldt, D. C. *et al.* Comprehensive molecular portraits of human breast tumours. *Nature* **490**, 61-70, doi:10.1038/nature11412 (2012).

2 Bell, D. *et al.* Integrated genomic analyses of ovarian carcinoma. *Nature* **474**, 609-615, doi:10.1038/nature10166 (2011).

3 Razavi, P. *et al.* The Genomic Landscape of Endocrine-Resistant Advanced Breast Cancers. *Cancer Cell* **34**, 427-438.e426, doi:10.1016/j.ccell.2018.08.008 (2018).
