# Supplementary material for: A transcription-based mechanism for oncogenic β-catenin-induced lethality in BRCA1/2-deficient cells
Source: Nat Commun. 2021 Aug 13;12:4919. doi: 10.1038/s41467-021-25215-0 (PMC8363664; doi:10.1038/s41467-021-25215-0)
Supplement: Supplementary file 3 — Description of Additional Supplementary Files [file 41467_2021_25215_MOESM3_ESM.pdf]

Description of Additional Supplementary Files

File Name: Supplementary Data 1
Description: Distribution of constitutive and β-catenin-induced replication origins in BRCA2-deficient H1299 cells.
