# Supplementary material for: A transcription-based mechanism for oncogenic β-catenin-induced lethality in BRCA1/2-deficient cells
Source: Nat Commun. 2021 Aug 13;12:4919. doi: 10.1038/s41467-021-25215-0 (PMC8363664; doi:10.1038/s41467-021-25215-0)
Supplement: Supplementary file 5 — Reporting Summary [file 41467_2021_25215_MOESM5_ESM.pdf]

nature portfolio

Corresponding author(s): M. Tarsounas

Last updated by author(s): Jul 1, 2021

Reporting Summary

Nature Portfolio wishes to improve the reproducibility of the work that we publish. This form provides structure for consistency and transparency in reporting. For further information on Nature Portfolio policies, see our Editorial Policies and the Editorial Policy Checklist.

Statistics

For all statistical analyses, confirm that the following items are present in the figure legend, table legend, main text, or Methods section.

n/a	Confirmed	
☐	☒	The exact sample size (*n*) for each experimental group/condition, given as a discrete number and unit of measurement
☐	☒	A statement on whether measurements were taken from distinct samples or whether the same sample was measured repeatedly
☐	☒	The statistical test(s) used AND whether they are one- or two-sided<br>*Only common tests should be described solely by name; describe more complex techniques in the Methods section.*
☒	☐	A description of all covariates tested
☐	☒	A description of any assumptions or corrections, such as tests of normality and adjustment for multiple comparisons
☐	☒	A full description of the statistical parameters including central tendency (e.g. means) or other basic estimates (e.g. regression coefficient) AND variation (e.g. standard deviation) or associated estimates of uncertainty (e.g. confidence intervals)
☐	☒	For null hypothesis testing, the test statistic (e.g. *F*, *t*, *r*) with confidence intervals, effect sizes, degrees of freedom and *P* value noted<br>*Give P values as exact values whenever suitable.*
☒	☐	For Bayesian analysis, information on the choice of priors and Markov chain Monte Carlo settings
☒	☐	For hierarchical and complex designs, identification of the appropriate level for tests and full reporting of outcomes
☒	☐	Estimates of effect sizes (e.g. Cohen's *d*, Pearson's *r*), indicating how they were calculated

Our web collection on statistics for biologists contains articles on many of the points above.

Software and code

Policy information about availability of computer code

Data collection	Omega V5.10 R2, Omega Data Analysis V3.02 R2, StepOne software v2.3, BD Cell Quest Pro v6
Data analysis	MS Excel 16.5, Graphpad Prism 9, FlowJo V9.4, Metamorph v7.5.1.0 , Image J v2.1.0/1.53c, R package DESeq2 version 1.26.0, Gene Set Enrichment Analysis (GSEA) software v7.4 with the Molecular Signatures Database collection (Hallmark gene sets), HISAT2 2.1.0, Picard MarkDuplicates tool 4.0. 1.1, featureCounts 2.0.1

For manuscripts utilizing custom algorithms or software that are central to the research but not yet described in published literature, software must be made available to editors and reviewers. We strongly encourage code deposition in a community repository (e.g. GitHub). See the Nature Portfolio guidelines for submitting code & software for further information.

Data

Policy information about availability of data

All manuscripts must include a data availability statement. This statement should provide the following information, where applicable:

- Accession codes, unique identifiers, or web links for publicly available datasets
- A description of any restrictions on data availability
- For clinical datasets or third party data, please ensure that the statement adheres to our policy

Source data are provided with this paper. Sequencing data area accessible at the Gene Expression Omnibus (GEO) repository, under accession number GSE153736 (https://www.ncbi.nlm.nih.gov/geo/query/acc.cgi?acc=GSE153736). The TCGA and MSK data are available in the cBioPortal for Cancer Genomics database (https://www.cbioportal.org).

Field-specific reporting

Please select the one below that is the best fit for your research. If you are not sure, read the appropriate sections before making your selection.

☒ Life sciences ☐ Behavioural & social sciences ☐ Ecological, evolutionary & environmental sciences

For a reference copy of the document with all sections, see nature.com/documents/nr-reporting-summary-flat.pdf

Life sciences study design

All studies must disclose on these points even when the disclosure is negative.

Sample size	Experiments were performed as biological replicates with at least two independent replicates as indicated in each figure legend. Sample size was determined to obtain measurable statistical power, , which was established from previous published works.
Data exclusions	No data points were excluded
Replication	For all experiments the number of biological replicates are indicated and reproduced the data shown in the figure.
Randomization	In each experiment, a parental culture of cells was split into individual cultures randomly. Each culture received drug treatment or siRNA without predetermination.
Blinding	Microscopy pictures were blinded before quantification. Clonogenic cell survival assay replicates were blinded to the investigators. For the remaining experiments the researchers were aware of the treatment conditions while acquiring and analysing the experiments. As viability, proliferation assays, and FACS data collection are performed in an automated unbiased way blinding was not necessary.

Reporting for specific materials, systems and methods

We require information from authors about some types of materials, experimental systems and methods used in many studies. Here, indicate whether each material, system or method listed is relevant to your study. If you are not sure if a list item applies to your research, read the appropriate section before selecting a response.

Materials & experimental systems

n/a	Involved in the study	
☐	☒	Antibodies
☐	☒	Eukaryotic cell lines
☒	☐	Palaeontology and archaeology
☒	☐	Animals and other organisms
☒	☐	Human research participants
☒	☐	Clinical data
☒	☐	Dual use research of concern

Methods

n/a	Involved in the study	
☒	☐	ChIP-seq
☐	☒	Flow cytometry
☒	☐	MRI-based neuroimaging

Antibodies

Antibodies used

Immunoblotting mouse monoclonal antibodies:
BRCA1 (1:1,000, Calbiochem, OP92),
BRCA2 (1:1,000, Calbiochem, OP95),
CDC6 (1:1,000, Santa Cruz, sc-9964),
CHK1 [G-4] (1:1,00, Santa Cruz, SC-8408),
Cyclin E [HE12] (1:1,000, Santa Cruz, sc-247),
FLAG [M2] (1:5,000, Stratagene, 200472-21),
GAPDH [6C5] (1:30,000, Novus Biologicals, NB600-502),
RPA [9H8] (1:500, Abcam, ab2175);

Immunoblotting rabbit polyclonal antibodies:
B-catenin (1:1,000, Cell Signaling, 9562S),
cyclin D1 (1:1,000, Cell Signaling, 2922S),
phosphorylated Ser33/Ser34/Thr41 B-catenin (1:500, Cell Signaling, 9561),
phosphorylated Ser317 CHK1 (1:2,000, Cell Signaling, 2344),
phosphorylated Ser345 CHK1 (1:1,000, Cell Signaling, 2341),
GSK3α/β (1:1,000, Cell Signaling, 5676S),
phosphorylated Y279/Y216 GSK3α/β (1:1,000, Abcam, AB75745),
cleaved PARP [Asp214] [D64E10] (1:1,000, Cell Signaling, 5625S),
PARP [46D11] (1:1,000, Cell Signaling, 9532S),
phosphorylated Ser33 RPA (1:1,000, Bethyl Laboratories, A300-246A-1),

SMC1 [BL308] (1:10,000, Bethyl Laboratories, A300-055A),
p21 Waf1/Cip1 [12D1] (1:1,000, Cell Signalling, 2947S).

Immunoblotting secondary antibodies:
anti-mouse Horseradish peroxidase (HRP)-conjugated secondary antibody (1:5,000, DAKO, P0447)
anti-rabbit Horseradish peroxidase (HRP)-conjugated secondary antibody (1:5,000, DAKO, P0448 respectively)

Immunofluorescence mouse monoclonal antibody:
BrdU (1:100, Becton Dickinson, 347580)

Immunoflourescence rabbit polyclonal antibody:
53BP1 (1:5,000, Novus, NB100-304),
nucleolin (1:1,000, Abcam, ab50279).

Monoclonal S9.6 antibody (1:1,000, hybridoma cell line HB-8730) was used for immunofluorescence and DRIP analysis.

Immunofluorescnce secondary antibodies:
anti-rabbit Alexa Fluor 488 (1:1,000, Invitrogen, A11008)
anti-mouse Alexa Fluor 594 (1:1,000, Invitrogen, A11005)
anti-rat Alexa Fluor 555 (1:300; Invitrogen, A21434)
anti-mouse Alexa Fluor 488 (1:300; Invitrogen, A11001)

Validation

Validation statements for these antibodies for use with human cells from the manufacturer's websites:
Immunoblotting mouse monoclonal antibodies:
BRCA1 (1:1,000, Calbiochem, OP92), https://www.sigmaaldrich.com/GB/en/product/mm/op92
BRCA2 (1:1,000, Calbiochem, OP95), https://www.sigmaaldrich.com/GB/en/product/mm/op95
CDC6 (1:1,000, Santa Cruz, sc-9964), https://www.scbt.com/p/cdc6-antibody-180-2
CHK1 [G-4] (1:1,00, Santa Cruz, SC-8408), https://www.scbt.com/p/chk1-antibody-g-4
Cyclin E [HE12] (1:1,000, Santa Cruz, sc-247), https://www.scbt.com/p/cyclin-e-antibody-he12
FLAG [M2] (1:5,000, Stratagene, 200472-21), single band on WB only detected upon inducible expression of tagged protein.
GAPDH [6C5] (1:30,000, Novus Biologicals, NB600-502), https://www.novusbio.com/products/gapdh-antibody-6c5cc_nb600-502
RPA [9H8] (1:500, Abcam, ab2175), https://www.abcam.com/rpa32rpa2-antibody-9h8-ab2175.html

Immunoblotting rabbit polyclonal antibodies:
B-catenin (1:1,000, Cell Signaling, 9562S), https://www.cellsignal.co.uk/products/primary-antibodies/b-catenin-antibody/9562
cyclin D1 (1:1,000, Cell Signaling, 2922S), https://www.cellsignal.co.uk/products/primary-antibodies/cyclin-d1-antibody/2922
phosphorylated Ser33/Ser34/Thr41 B-catenin (1:500, Cell Signaling, 9561),
phosphorylated Ser317 CHK1 (1:2,000, Cell Signaling, 2344), https://www.cellsignal.co.uk/products/primary-antibodies/phospho-b-catenin-ser33-37-thr41-antibody/9561
phosphorylated Ser345 CHK1 (1:1,000, Cell Signaling, 2341), https://www.cellsignal.co.uk/products/primary-antibodies/phospho-chk1-ser345-antibody/2341
GSK3α/β (1:1,000, Cell Signaling, 5676S), https://www.cellsignal.co.uk/products/primary-antibodies/gsk-3a-b-d75d3-rabbit-mab/5676
phosphorylated Y279/Y216 GSK3α/β (1:1,000, Abcam, AB75745), https://www.abcam.com/gsk3-beta-phospho-y216--gsk3-alpha-phospho-y279-antibody-ab75745.html
cleaved PARP [Asp214] [D64E10] (1:1,000, Cell Signaling, 5625S), https://www.cellsignal.co.uk/products/primary-antibodies/cleaved-parp-asp214-d64e10-xp-rabbit-mab/5625
PARP [46D11] (1:1,000, Cell Signaling, 9532S), https://www.cellsignal.co.uk/products/primary-antibodies/parp-46d11-rabbit-mab/9532
phosphorylated Ser33 RPA (1:1,000, Bethyl Laboratories, A300-246A-1), https://www.bethyl.com/product/A300-246A/Phospho+RPA32+(S33)+Antibody
SMC1 [BL308] (1:10,000, Bethyl Laboratories, A300-055A), https://www.bethyl.com/product/A300-055A/SMC1+Antibody
p21 Waf1/Cip1 [12D1] (1:1,000, Cell Signalling, 2947S), https://www.cellsignal.co.uk/products/primary-antibodies/p21-waf1-cip1-12d1-rabbit-mab/2947

Immunofluorescence mouse monoclonal antibody:
BrdU (1:100, Becton Dickinson, 347580), https://www.bdbiosciences.com/en-us/products/reagents/flow-cytometry-reagents/clinical-discovery-research/single-color-antibodies-ruo-gmp/purified-mouse-anti-brdu.347580

Immunoflourescence rabbit polyclonal antibody:
53BP1 (1:5,000, Novus, NB100-304), https://www.novusbio.com/products/53bp1-antibody_nb100-304
nucleolin (1:1,000, Abcam, ab50279), https://www.abcam.com/nucleolin-antibody-ab50279.html

Monoclonal S9.6 antibody (1:1,000, hybridoma cell line HB-8730) was validated by performing a DRIP in parallel with the previous purification.

Eukaryotic cell lines

Policy information about cell lines

Cell line source(s)

Human non-small cell lung carcinoma H1299 cells (ATCC); human invasive ductal breast cancer MDA-MB-231 cells (ATCC); human colorectal adenocarcinoma DLD1 cells, parental and BRCA2-mutated (Horizon); human colorectal carcinoma HCT116 cells, parental and BRCA2-mutated (Ximbio, Cancer Research Technology); human cervical carcinoma HeLa cells carrying a

	DOX-inducible FLAG-tagged RNase H1 (gift from Prof. Karlene Cimprich, Stanford University, USA); human retinal pigment epithelial RPE1 cells transduced with hTERT and TP53-deleted, parental and BRCA1-deficient (a gift from Dr. Dan Durocher, University of Toronto, Canada); human osteosarcoma U2OS cells carrying a tetracycline-repressible (Tet-OFF) cassette (Thanos Halazonetis lab); human osteosarcoma U2OS cells (ATCC).
Authentication	Cell lines were authenticated using STR DNA profiling
Mycoplasma contamination	Cells tested negative for mycoplasma contamination
Commonly misidentified lines (See ICLAC register)	The used cell lines are not listed in the ICLAC database.

Flow Cytometry

Plots

Confirm that:

☐ The axis labels state the marker and fluorochrome used (e.g. CD4-FITC).

☒ The axis scales are clearly visible. Include numbers along axes only for bottom left plot of group (a 'group' is an analysis of identical markers).

☐ All plots are contour plots with outliers or pseudocolor plots.

☒ A numerical value for number of cells or percentage (with statistics) is provided.

Methodology

Sample preparation	To label replicated DNA, cells were incubated with 25 µM EdU for 30 min. Samples were collected by trypsinisation and fixed using 90% methanol. Incorporated EdU was detected using the Click-iT EdU Alexa Fluor 647 Flow Cytometry Assay Kit (C10634, Invitrogen) according to manufacturer's instructions. Cells were re-suspended in PBS containing 20 µg/mL propidium iodide (P4864, Sigma) and 400 µg/mL RNase A (12091-021, Invitrogen).
Instrument	Samples were processed using flow cytometry (342975, BD FACSCalibur, BD Biosciences).
Software	Cell Quest Pro was used to acquire data, 5,000- 10,000 events were analysed per condition using FlowJo software.
Cell population abundance	Forward scatter and side scatter were used to gate for live and single cells. EdU positive cells (%) were measured to determine the number of cells in S-phase.
Gating strategy	PI signal was used to gate for 2N and 4N populations. EdU signal was used to gate for cells in S-phase.

☒ Tick this box to confirm that a figure exemplifying the gating strategy is provided in the Supplementary Information.
