# Supplementary material for: A transcription-based mechanism for oncogenic β-catenin-induced lethality in BRCA1/2-deficient cells
Source: Nat Commun. 2021 Aug 13;12:4919. doi: 10.1038/s41467-021-25215-0 (PMC8363664; doi:10.1038/s41467-021-25215-0)
Supplement: Supplementary file 6 — Source Data [file 41467_2021_25215_MOESM6_ESM.zip › 283443_2_related_ms_5710975_qvsh23.pdf]

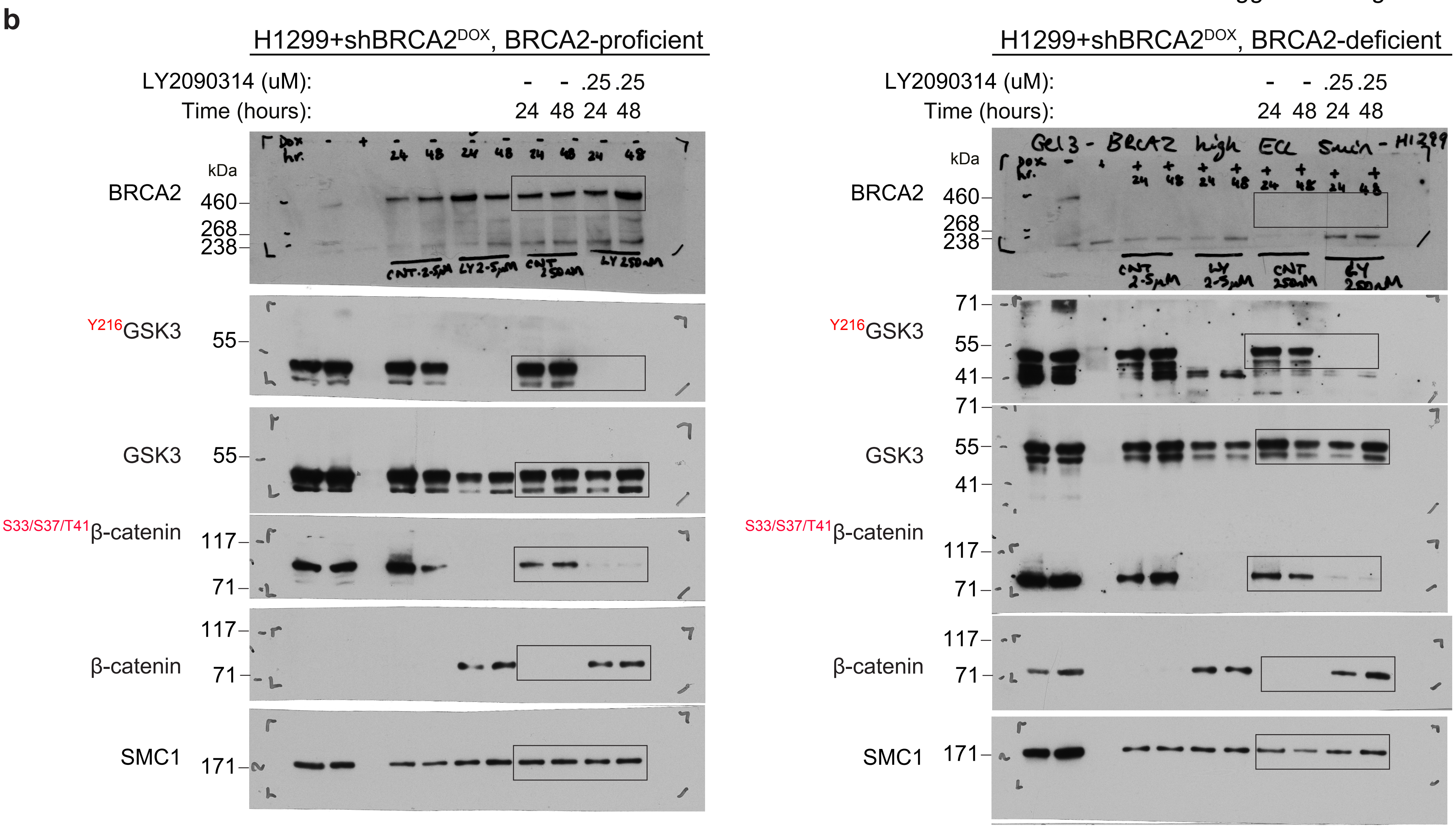
b
H1299+shBRCA2DOX, BRCA2-proficient
H1299+shBRCA2DOX, BRCA2-deficient
LY2090314 (uM): - - .25 .25
Time (hours): 24 48 24 48
kDa
BRCA2
460
268
238
Y216GSK3
55
41
71
GSK3
S33/S37/T41β-catenin
117
β-catenin
SMC1
171

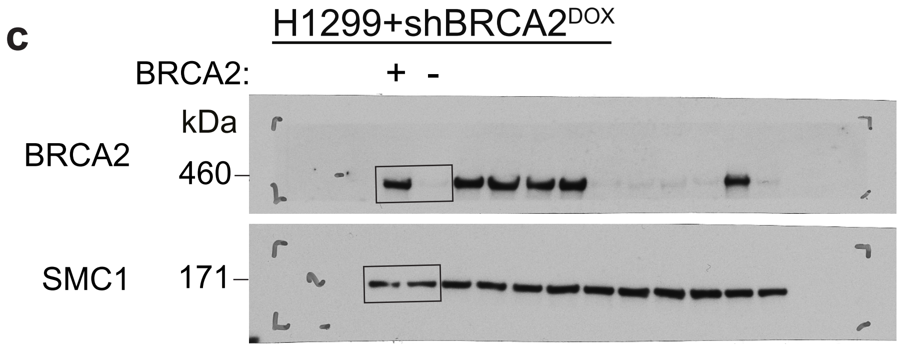
c
H1299+shBRCA2DOX
BRCA2:
+
-
kDa
BRCA2
460
SMC1
171

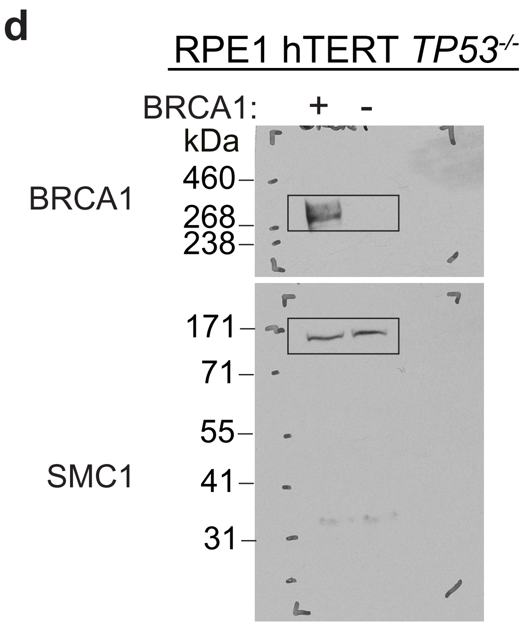
d
RPE1 hTERT TP53-/-
BRCA1:
+
-
kDa
460
268
238
BRCA1
171
71
55
41
31
SMC1

e

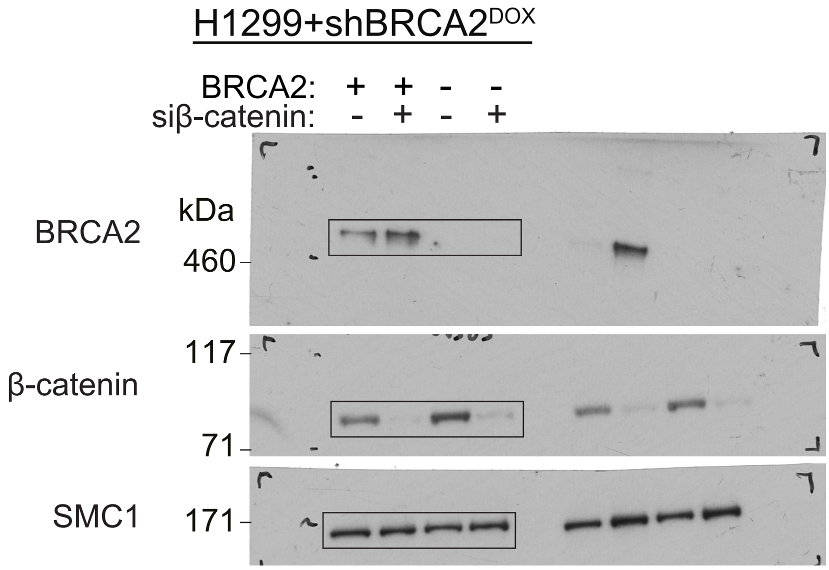
H1299+shBRCA2DOX
BRCA2: + + - -
siβ-catenin: - + - +
BRCA2
kDa
460
β-catenin
117
71
SMC1
171

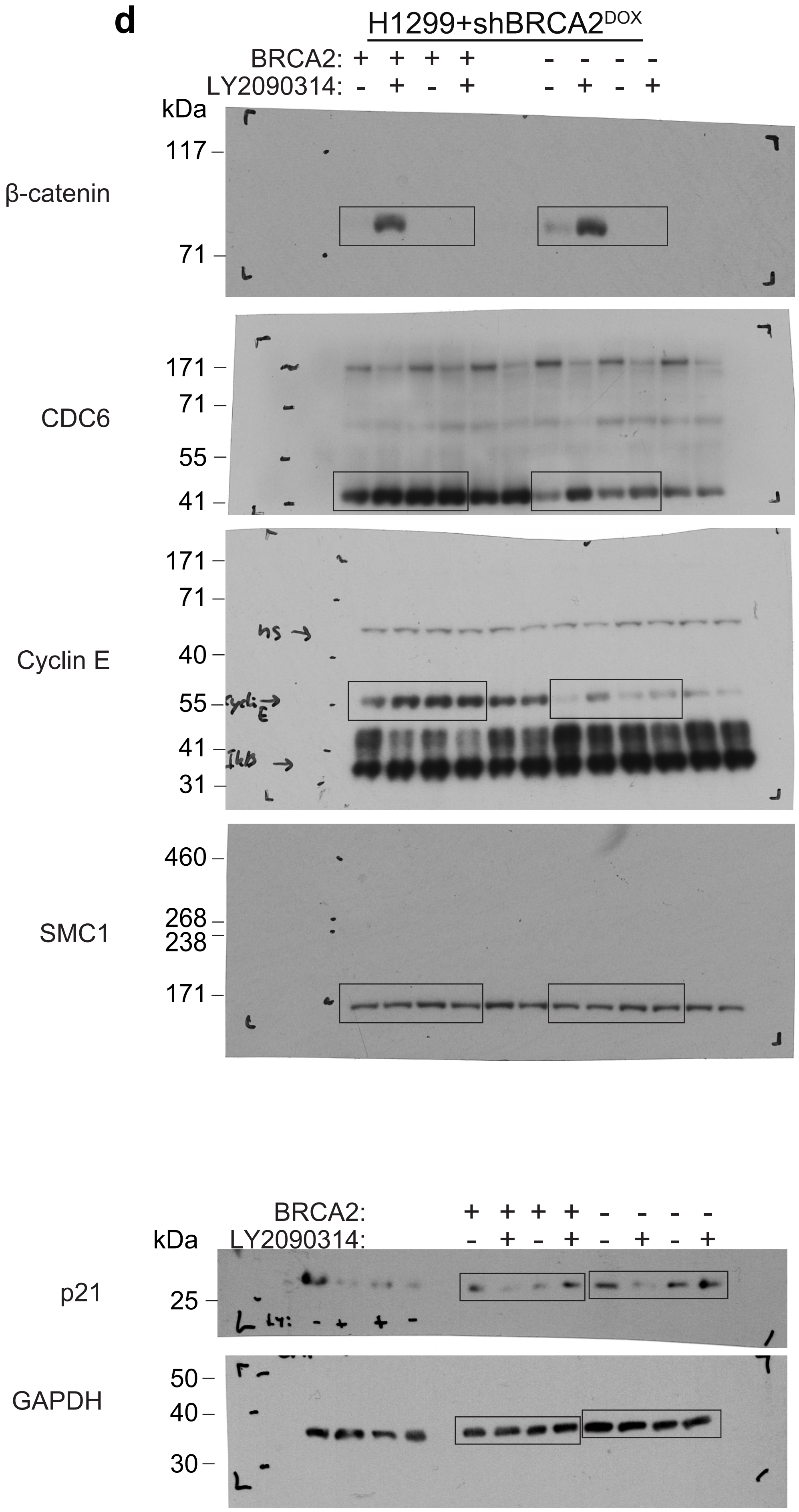
d
H1299+shBRCA2DOX
BRCA2: + + + + - - - -
LY2090314: - + - + - + - +
kDa
β-catenin
117
71
CDC6
171
71
55
41
Cyclin E
171
71
40
55
41
31
SMC1
460
268
238
171
BRCA2: + + + + - - - -
LY2090314: - + - + - + - +
kDa
p21
25
GAPDH
50
40
30

a

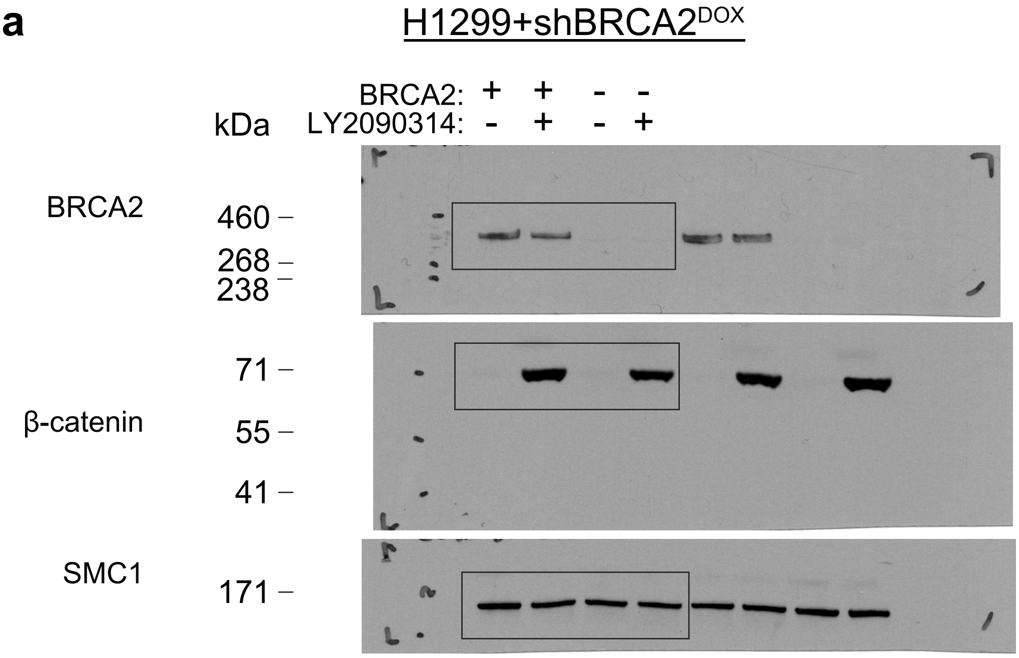
H1299+shBRCA2DOX
BRCA2: + + - -
LY2090314: - + - +
kDa
BRCA2
460
268
238
71
β-catenin
55
41
SMC1
171

b

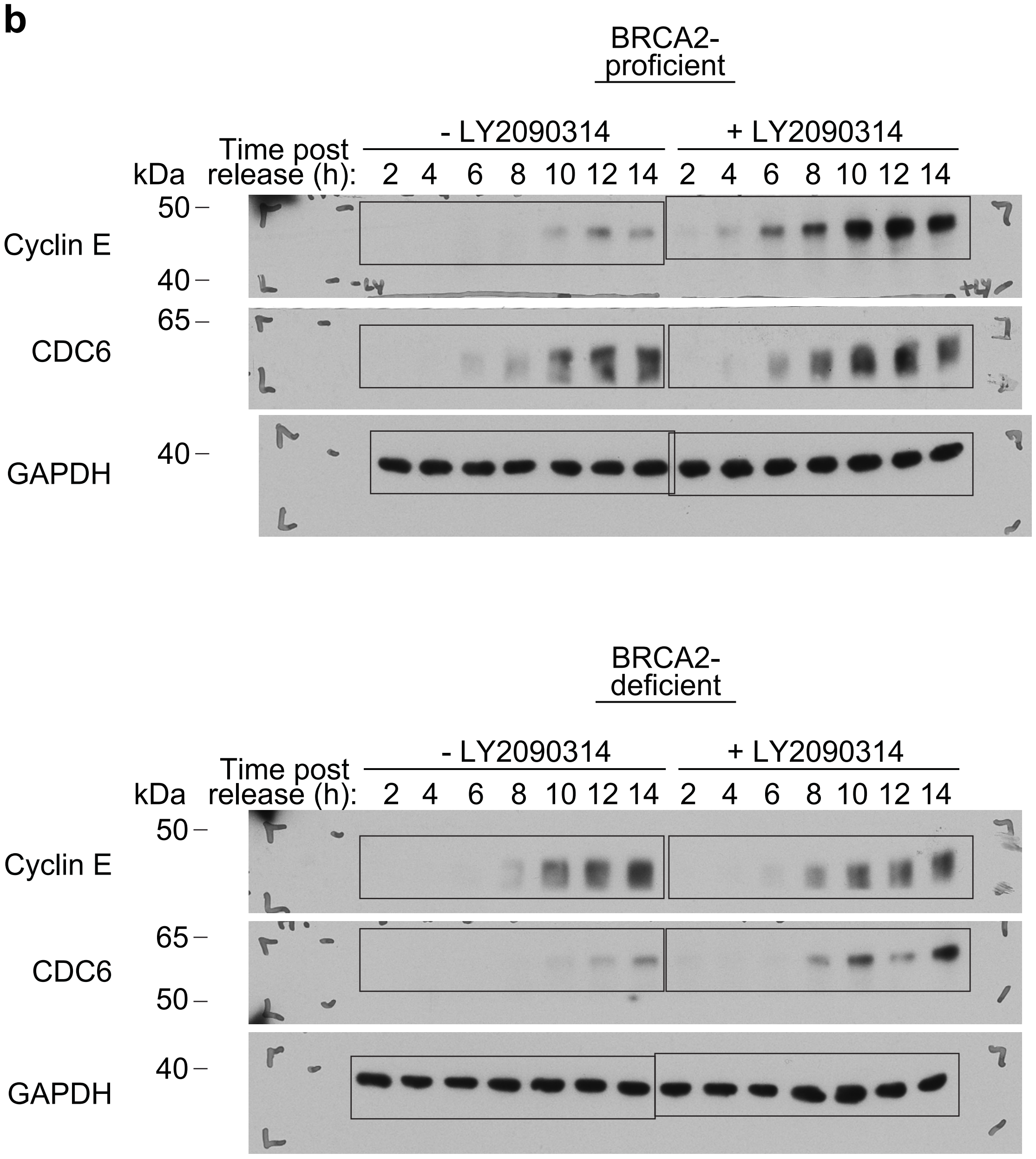
BRCA2-
proficient
- LY2090314
+ LY2090314
Time post
release (h): 2 4 6 8 10 12 14 2 4 6 8 10 12 14
kDa
50
40
65
40
Cyclin E
CDC6
GAPDH
BRCA2-
deficient
- LY2090314
+ LY2090314
Time post
release (h): 2 4 6 8 10 12 14 2 4 6 8 10 12 14
kDa
50
65
50
40
Cyclin E
CDC6
GAPDH

c

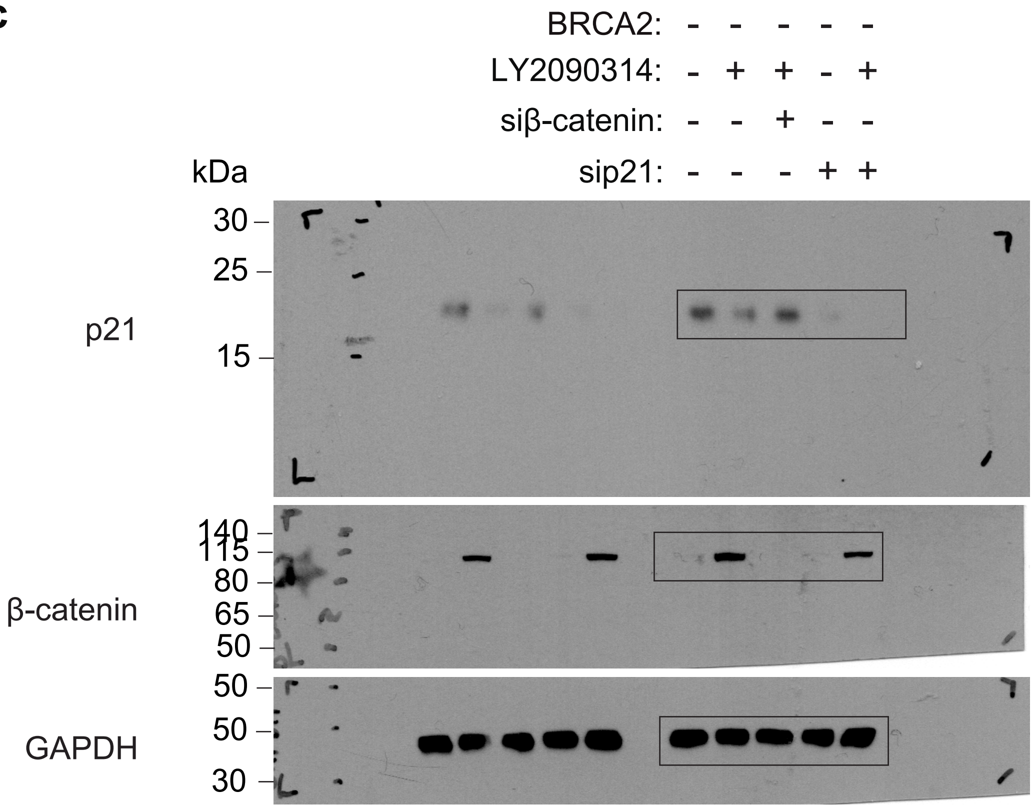
BRCA2: - - - - -
LY2090314: - + + - +
siβ-catenin: - - + - -
sip21: - - - + +
kDa
30
25
15
p21
140
115
80
65
50
β-catenin
50
50
30
GAPDH

d

H1299+shBRCA2^{DOX}

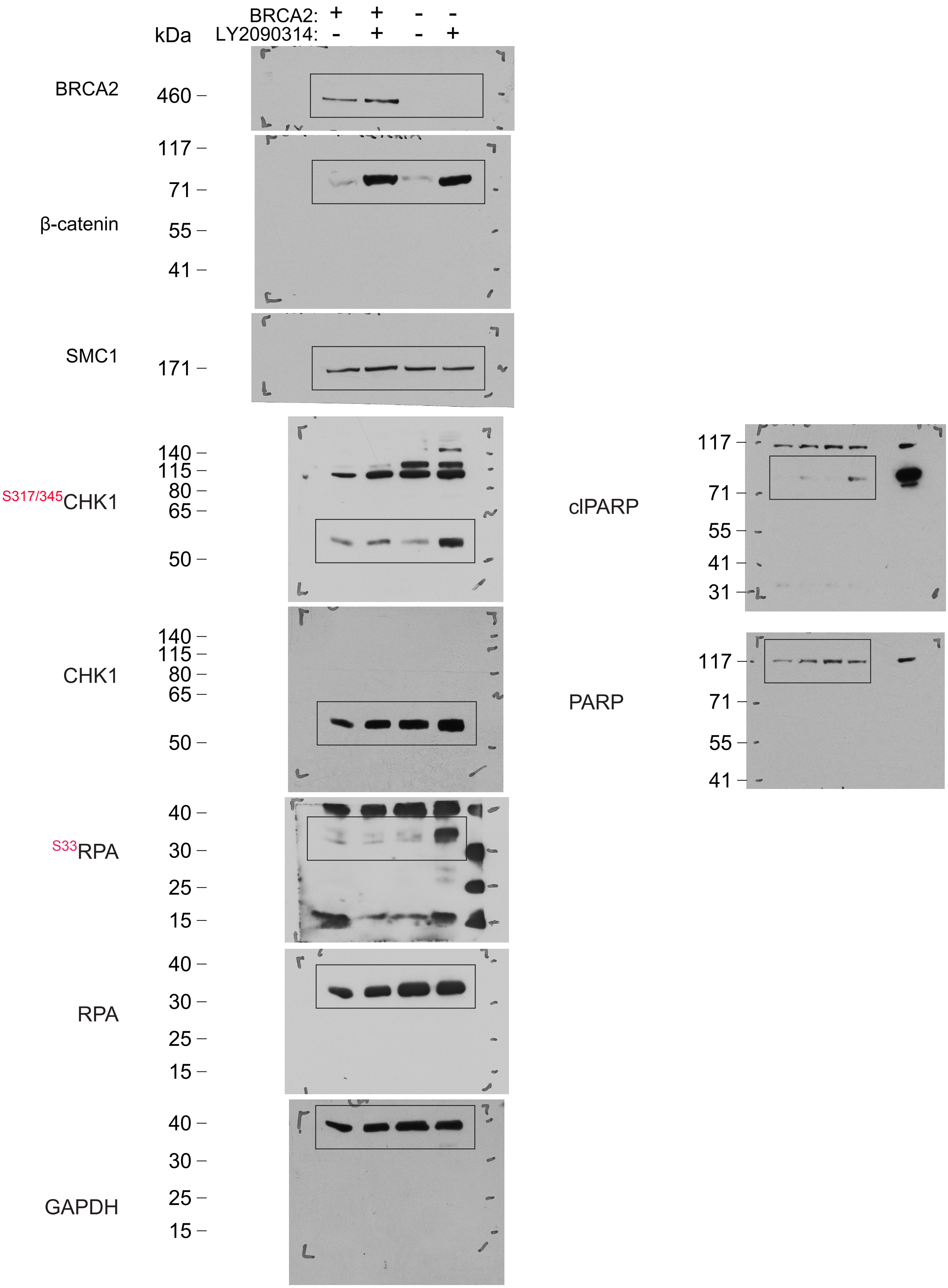

Dagg et al. - Figure 6

a

HeLa±siBRCA2

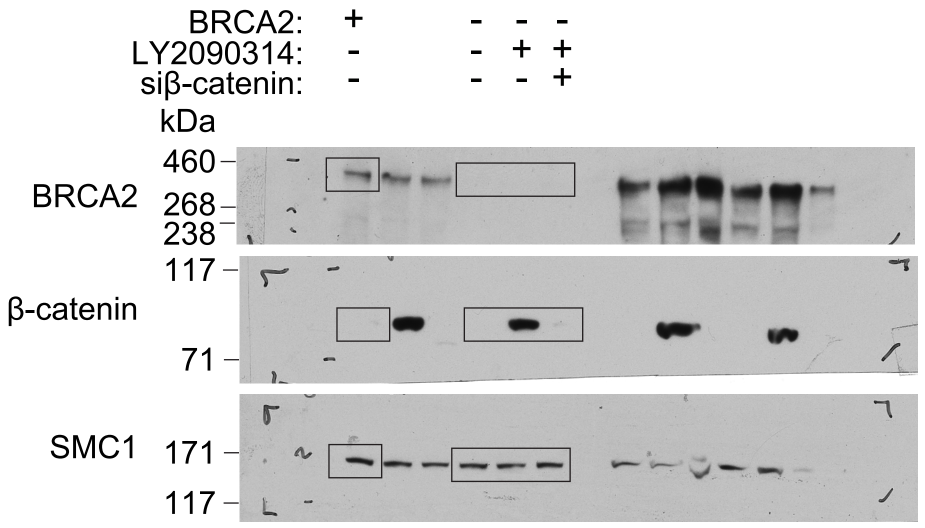

b

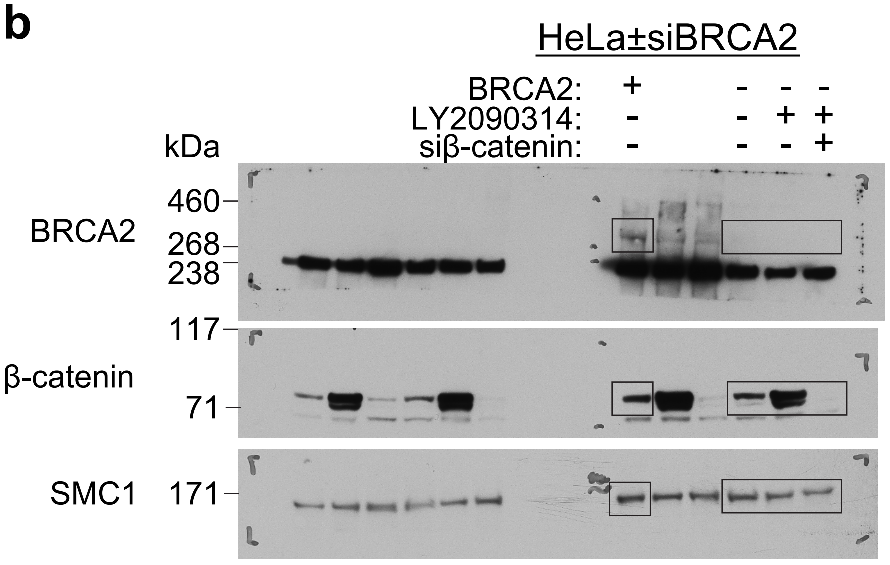
HeLa±siBRCA2
BRCA2: + - - -
LY2090314: - - + +
siβ-catenin: - - - +
kDa
BRCA2
460
268
238
117
β-catenin
71
SMC1
171

d

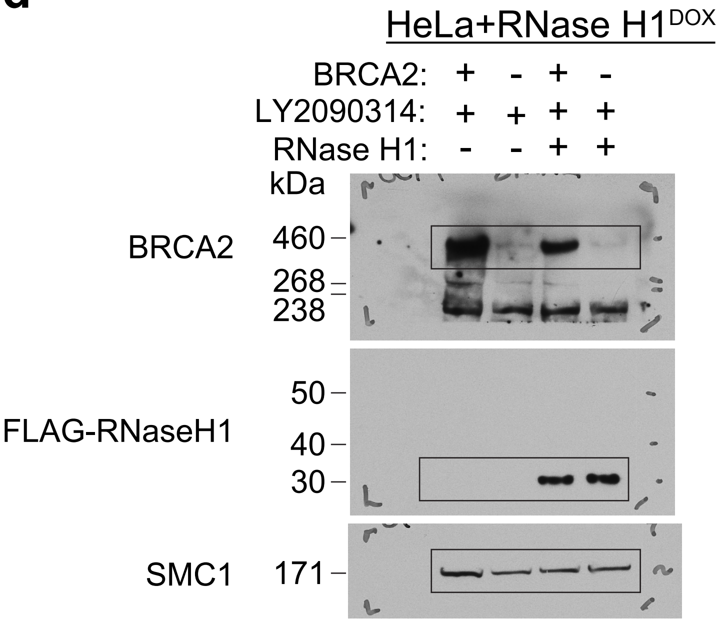
HeLa+RNase H1^DOX
BRCA2: + - + -
LY2090314: + + + +
RNase H1: - - + +
kDa
BRCA2
460
268
238
FLAG-RNaseH1
50
40
30
SMC1
171

a

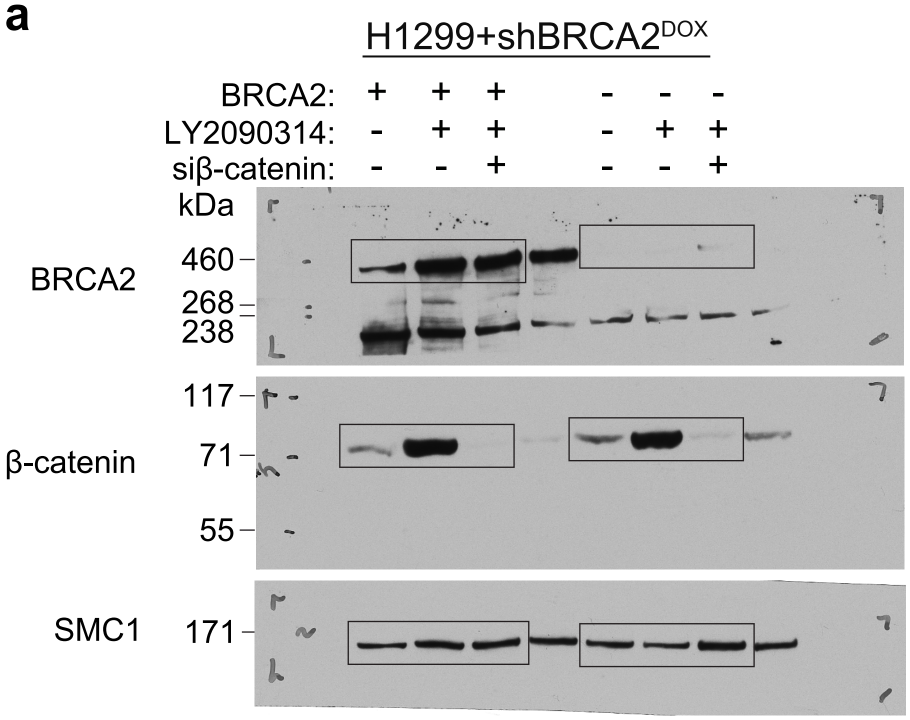
H1299+shBRCA2DOX
BRCA2: + + + - - -
LY2090314: - + + - + +
siβ-catenin: - - + - - +
kDa
460
268
238
BRCA2
117
71
55
β-catenin
171
SMC1

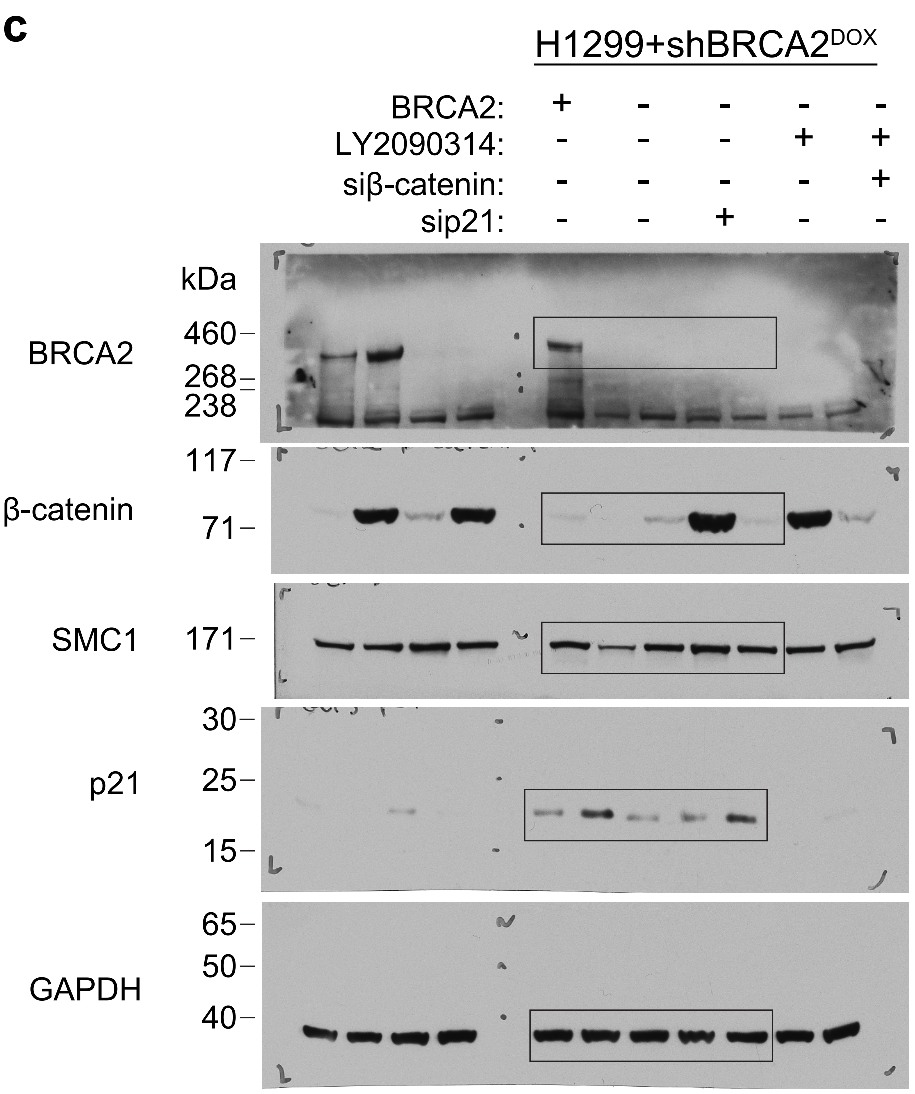
c
H1299+shBRCA2DOX
BRCA2: + - - - -
LY2090314: - - - + +
siβ-catenin: - - - - +
sip21: - - + - -
kDa
BRCA2
460
268
238
117
β-catenin
71
SMC1
171
30
p21
25
15
65
50
GAPDH
40

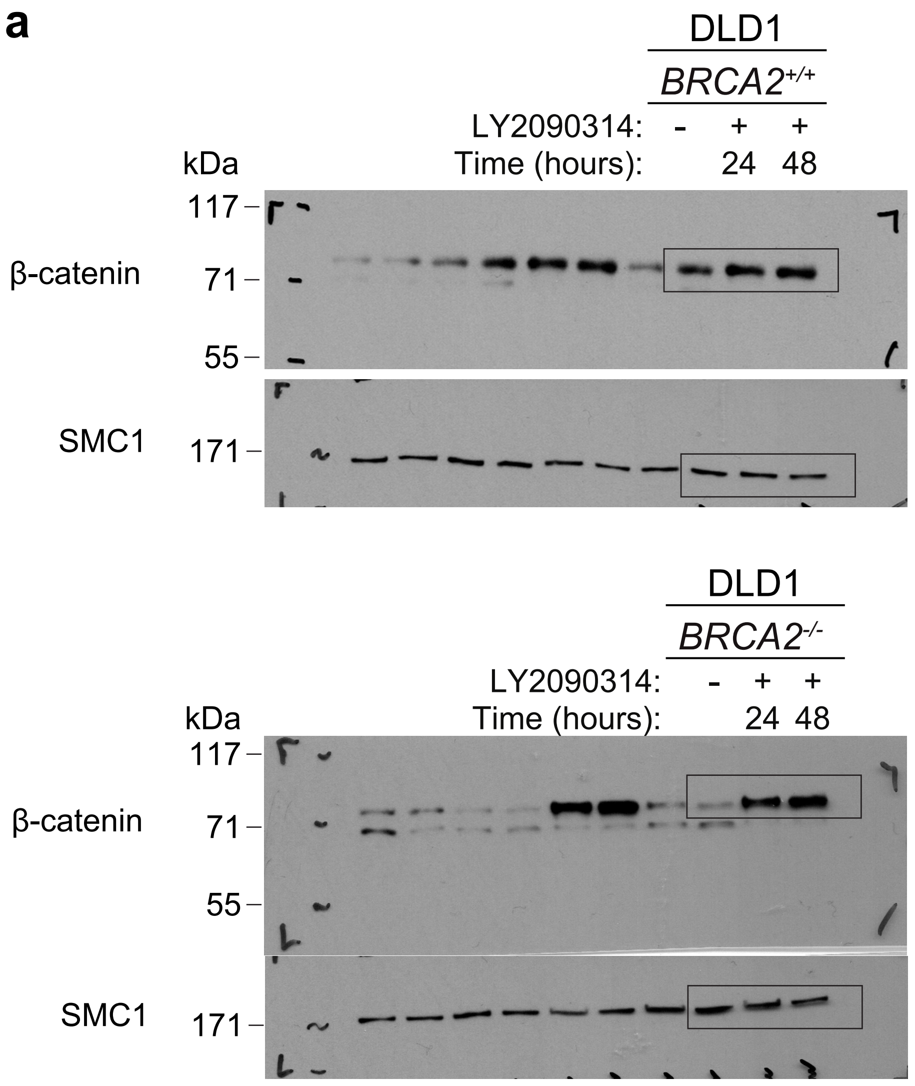
a
DLD1
BRCA2+/+
LY2090314: - + +
Time (hours): 24 48
kDa
117
71
55
β-catenin
SMC1
171
DLD1
BRCA2-/-
LY2090314: - + +
Time (hours): 24 48
kDa
117
71
55
β-catenin
SMC1
171

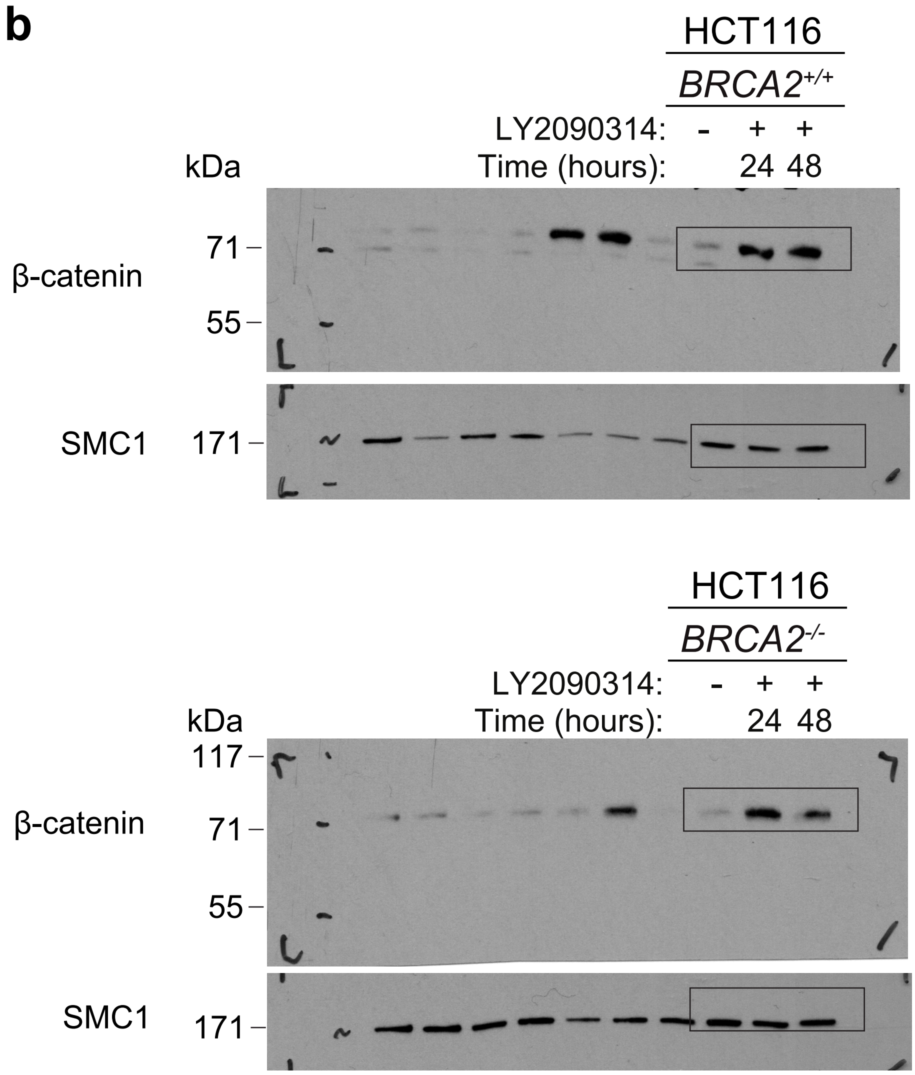
b
HCT116
BRCA2+/+
LY2090314: - + +
Time (hours): 24 48
kDa
β-catenin
71
55
SMC1
171
HCT116
BRCA2-/-
LY2090314: - + +
Time (hours): 24 48
kDa
117
β-catenin
71
55
SMC1
171

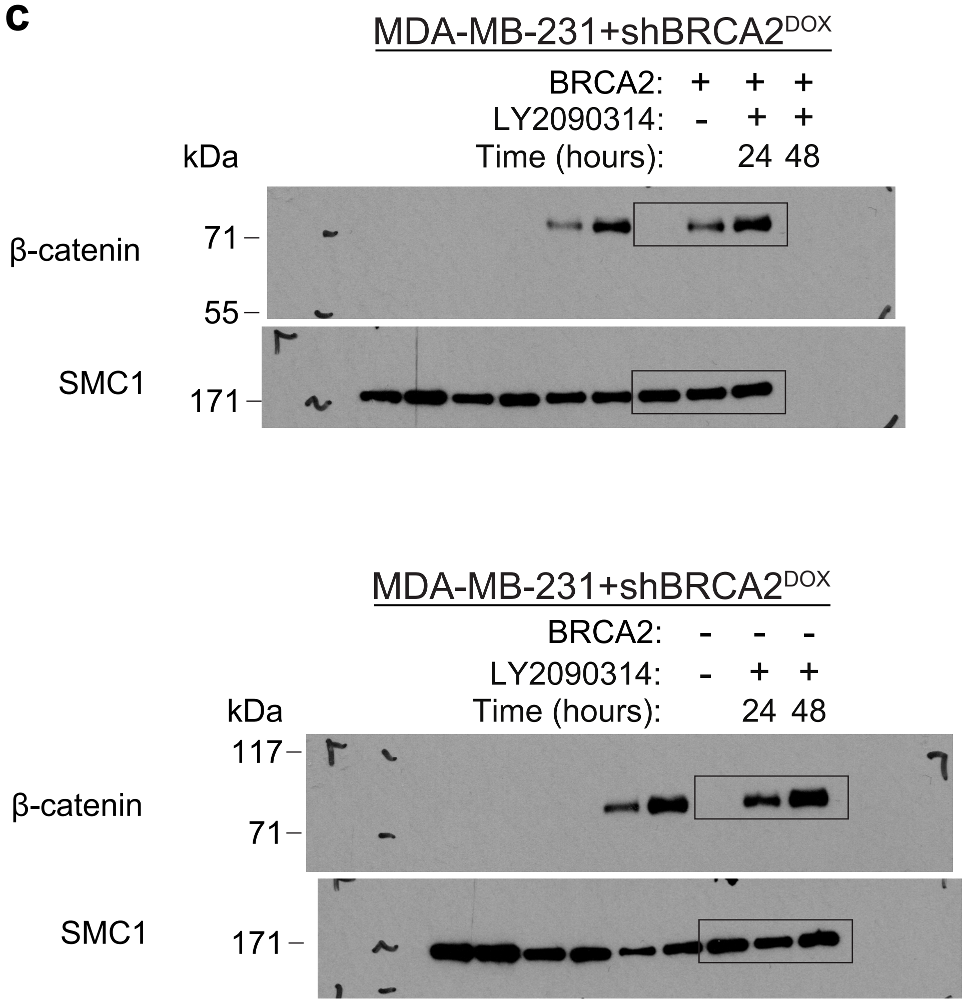
c
MDA-MB-231+shBRCA2DOX
BRCA2: + + +
LY2090314: - + +
Time (hours): 24 48
kDa
β-catenin
71
55
SMC1
171
MDA-MB-231+shBRCA2DOX
BRCA2: - - -
LY2090314: - + +
Time (hours): 24 48
kDa
117
β-catenin
71
SMC1
171

Dagg et al. - Supplementary Figure 2

d

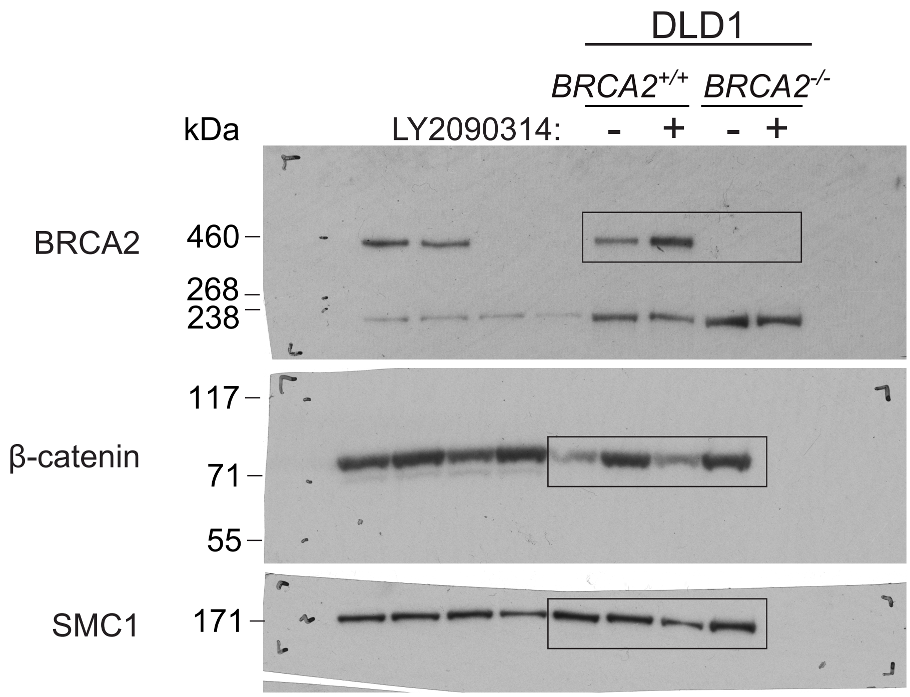

e

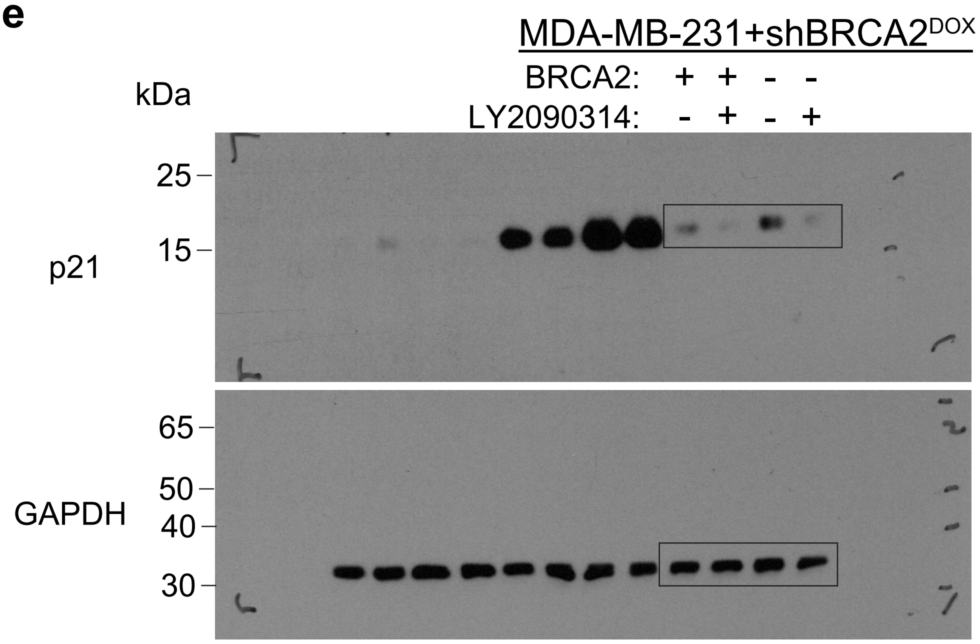
MDA-MB-231+shBRCA2DOX
BRCA2: + + - -
LY2090314: - + - +
kDa
25
15
p21
65
50
40
30
GAPDH

Dagg et al. - Supplementary Figure 2

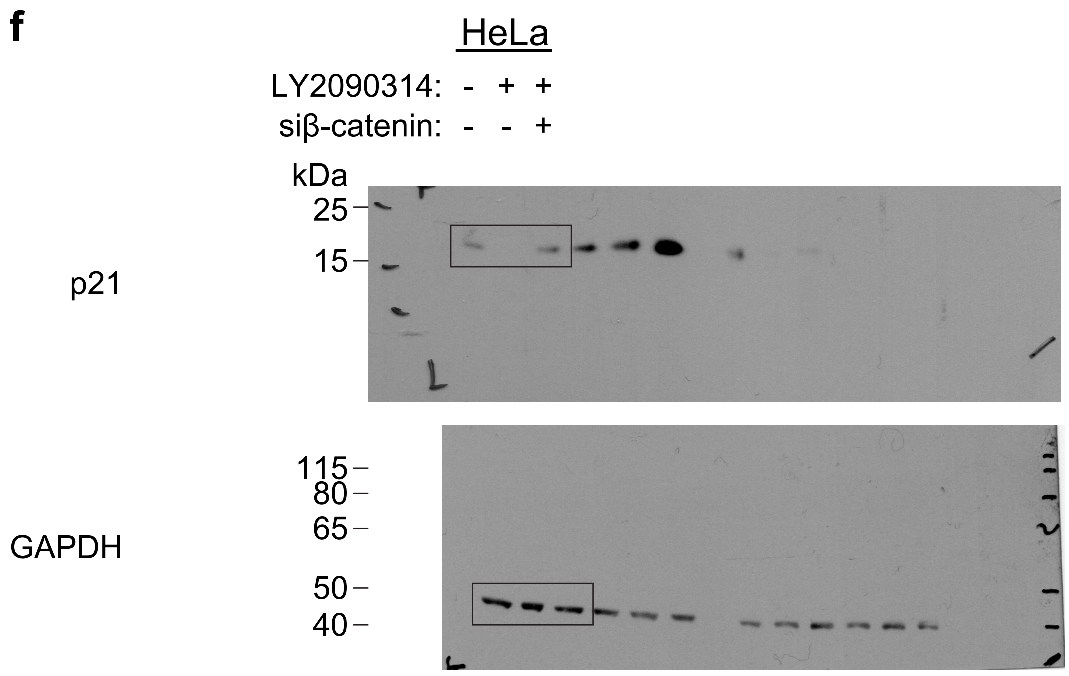

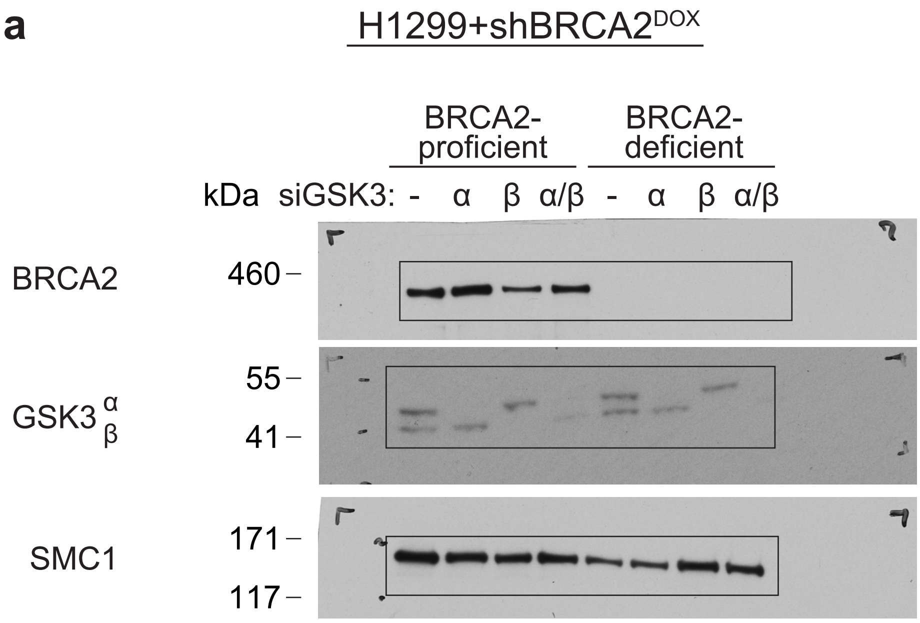

a
H1299+shBRCA2DOX
BRCA2-
proficient
BRCA2-
deficient
kDa siGSK3: - α β α/β - α β α/β
BRCA2
460
GSK3 α β
55
41
SMC1
171
117

a

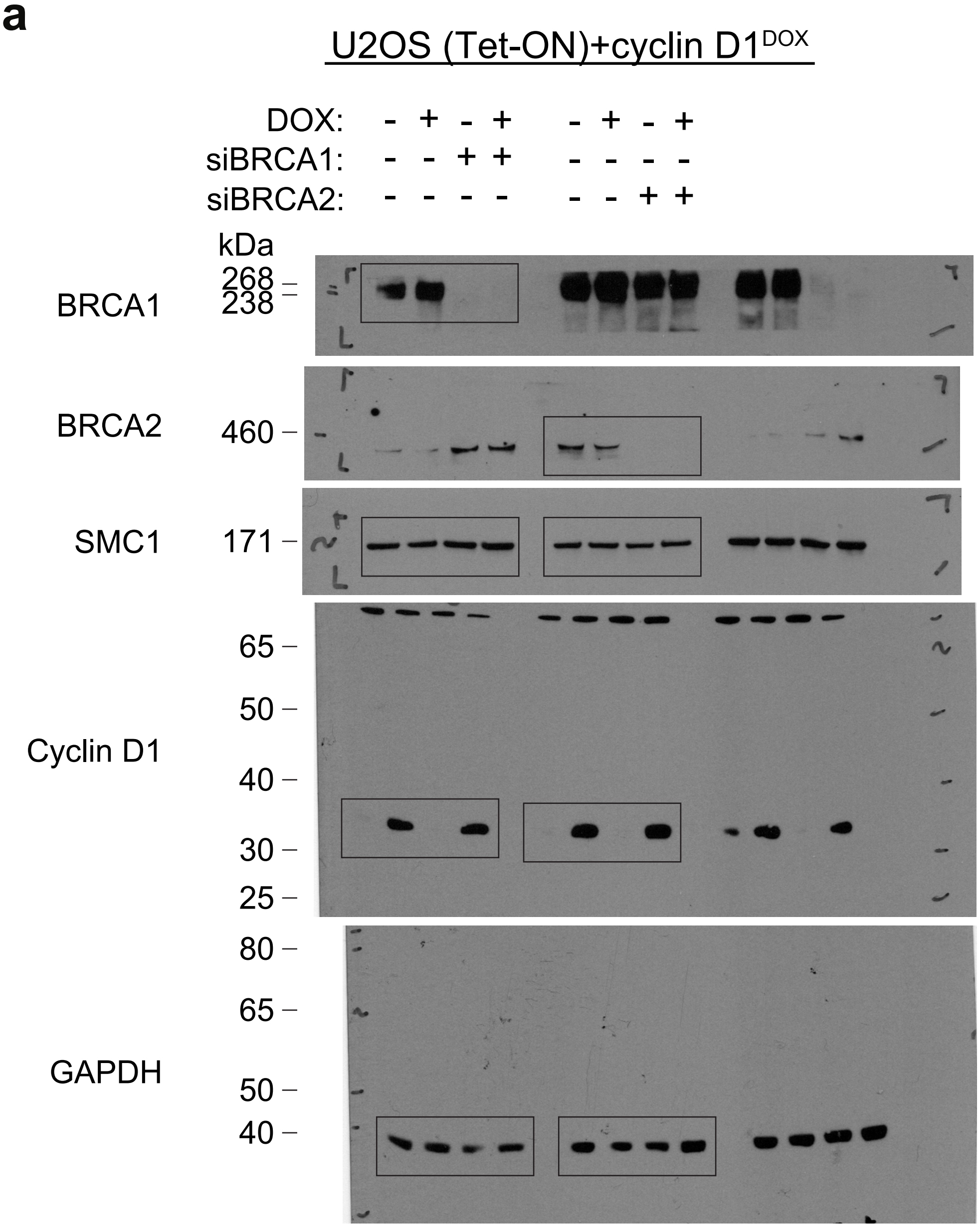
U2OS (Tet-ON)+cyclin D1DOX
DOX: - + - + - + - +
siBRCA1: - - + + - - - -
siBRCA2: - - - - - - + +
kDa
BRCA1
268
238
BRCA2
460
SMC1
171
Cyclin D1
65
50
40
30
25
GAPDH
80
65
50
40

Dagg et al. - Supplementary Figure 4

d

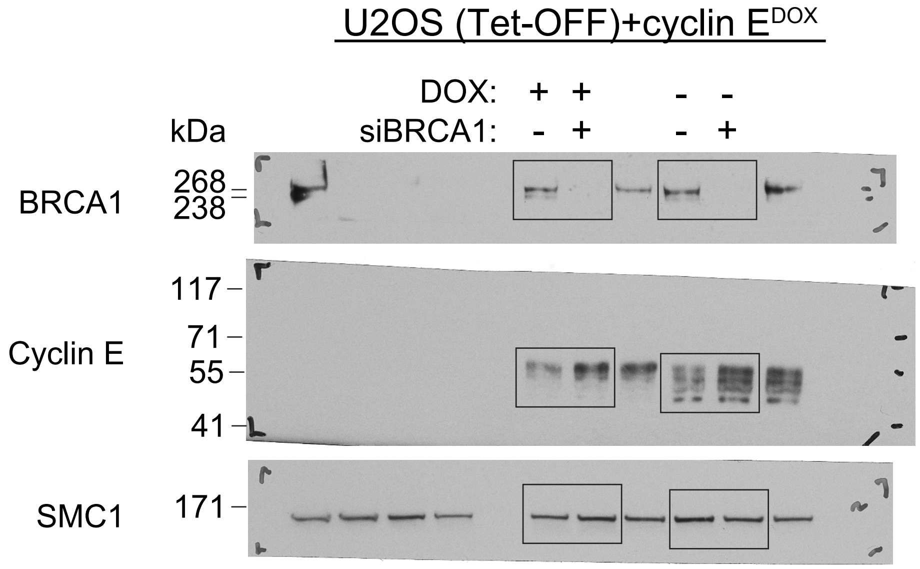

Dagg et al. - Supplementary Figure 6

b

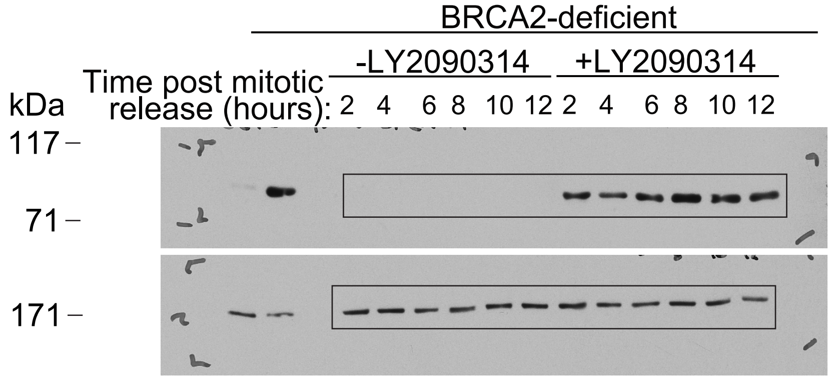

a

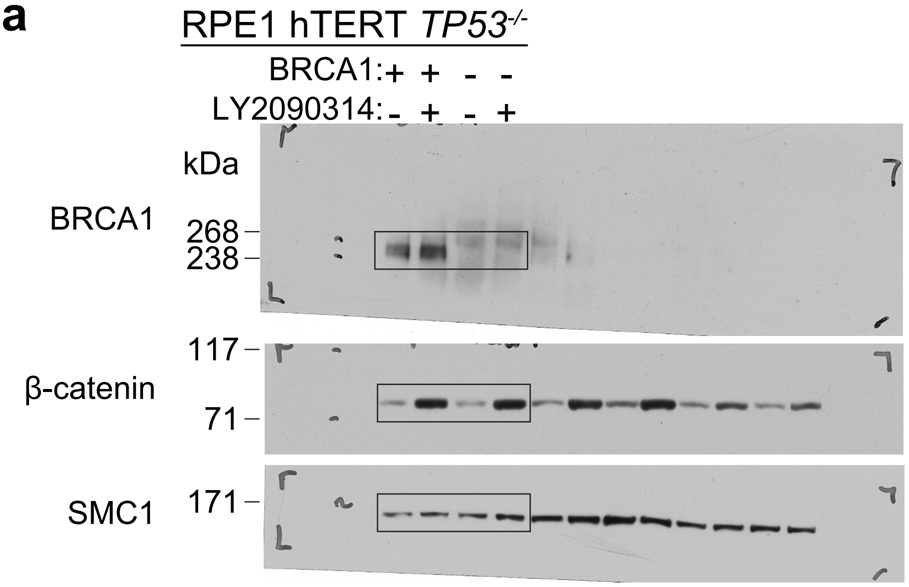
RPE1 hTERT TP53-/-
BRCA1: + + - -
LY2090314: - + - +
kDa
BRCA1
268
238
β-catenin
117
71
SMC1
171

b

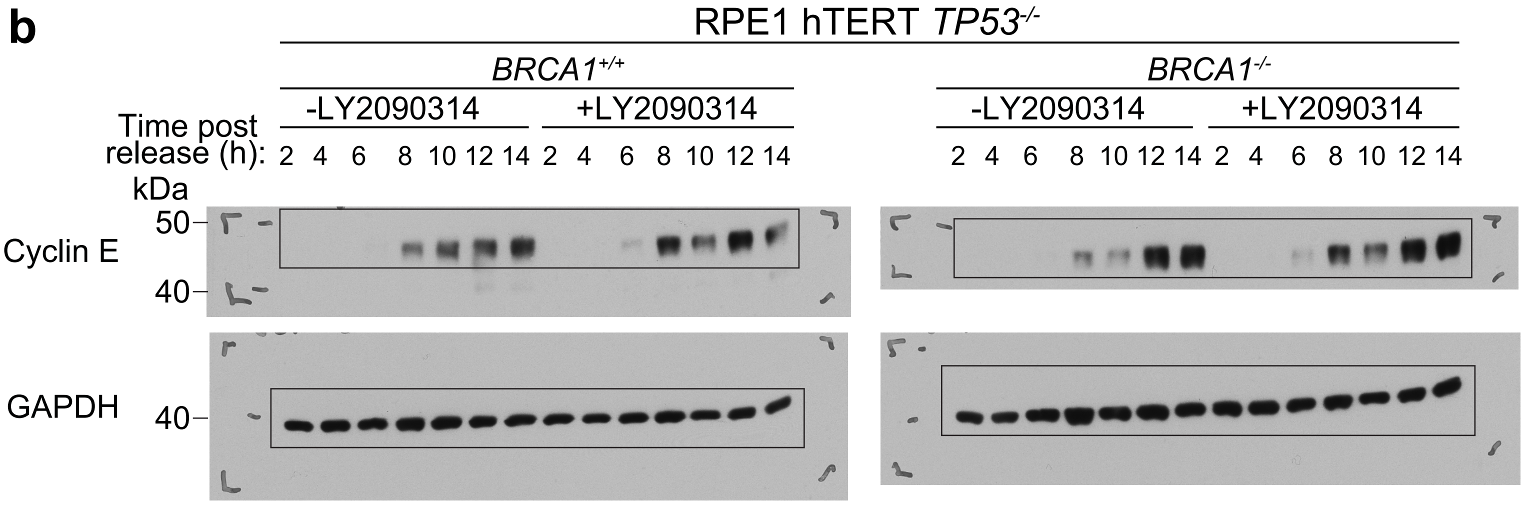
RPE1 hTERT TP53-/-
BRCA1+/+
BRCA1-/-
-LY2090314
+LY2090314
-LY2090314
+LY2090314
Time post release (h):
2 4 6 8 10 12 14 2 4 6 8 10 12 14
2 4 6 8 10 12 14 2 4 6 8 10 12 14
kDa
50
40
Cyclin E
GAPDH
40

a

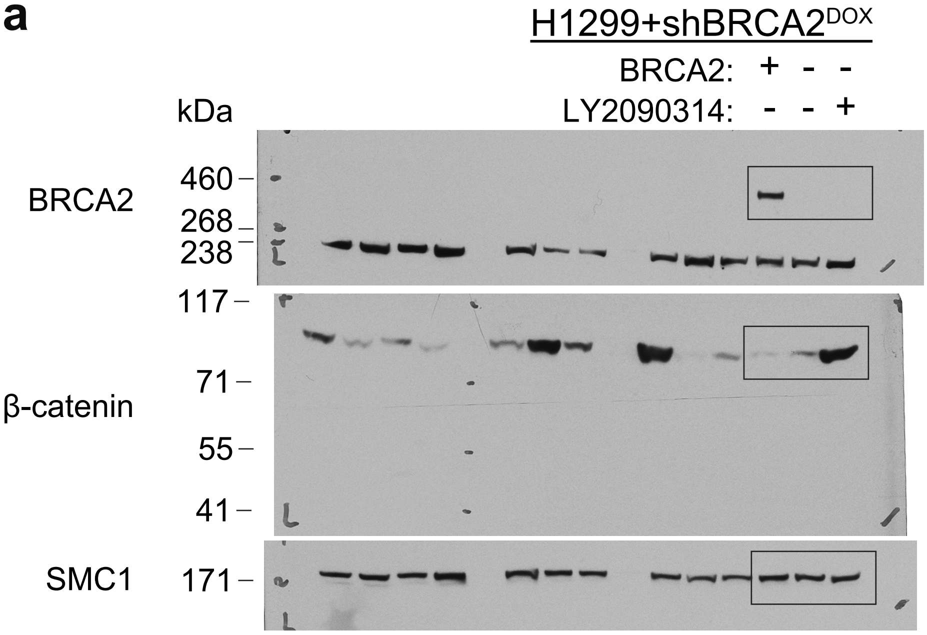
H1299+shBRCA2^DOX
BRCA2: + - -
LY2090314: - - +
kDa
BRCA2
460
268
238
117
β-catenin
71
55
41
SMC1
171

Dagg et al. - Supplementary Figure 10

c

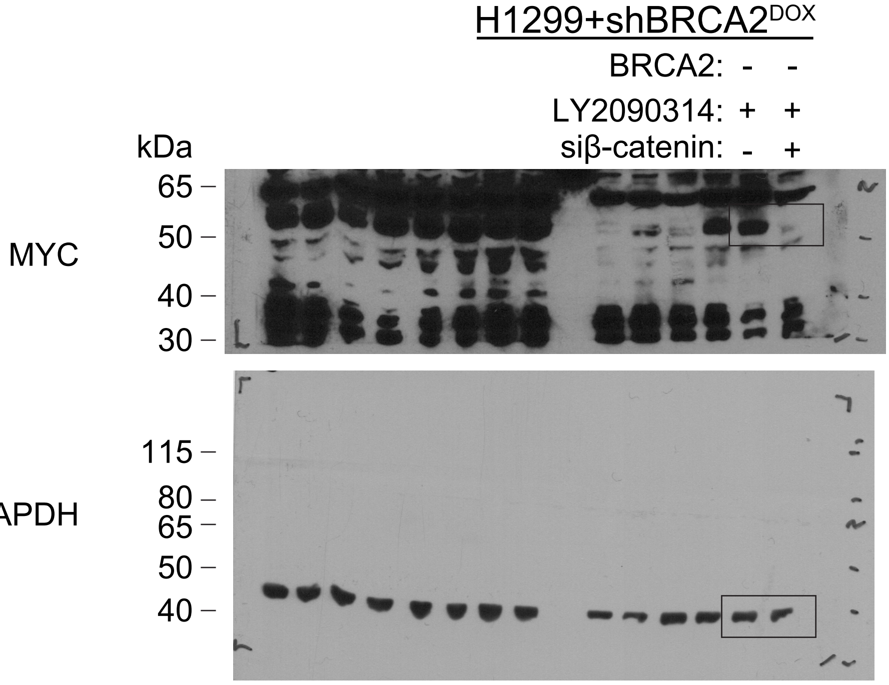

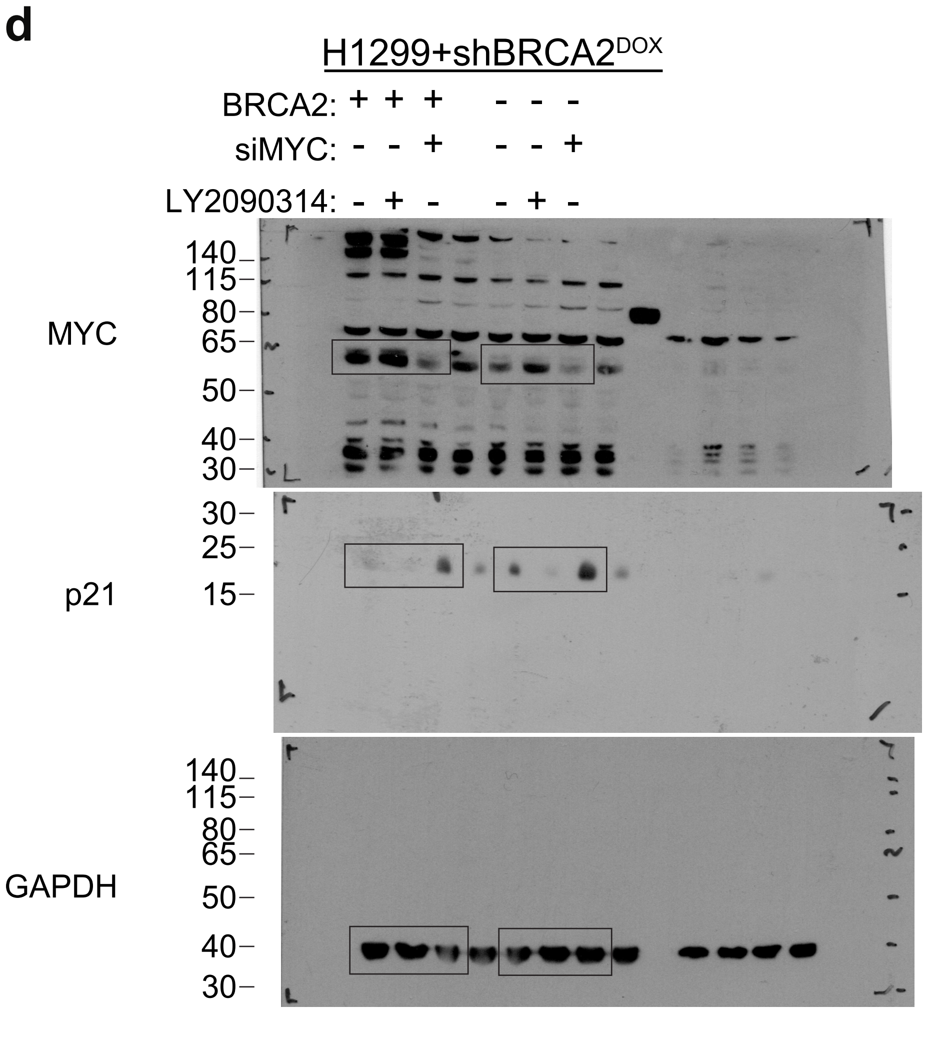
d
H1299+shBRCA2DOX
BRCA2: + + + - - -
siMYC: - - + - - +
LY2090314: - + - - + -
MYC
140
115
80
65
50
40
30
p21
30
25
15
GAPDH
140
115
80
65
50
40
30

Dagg et al. - Supplementary Figure 10

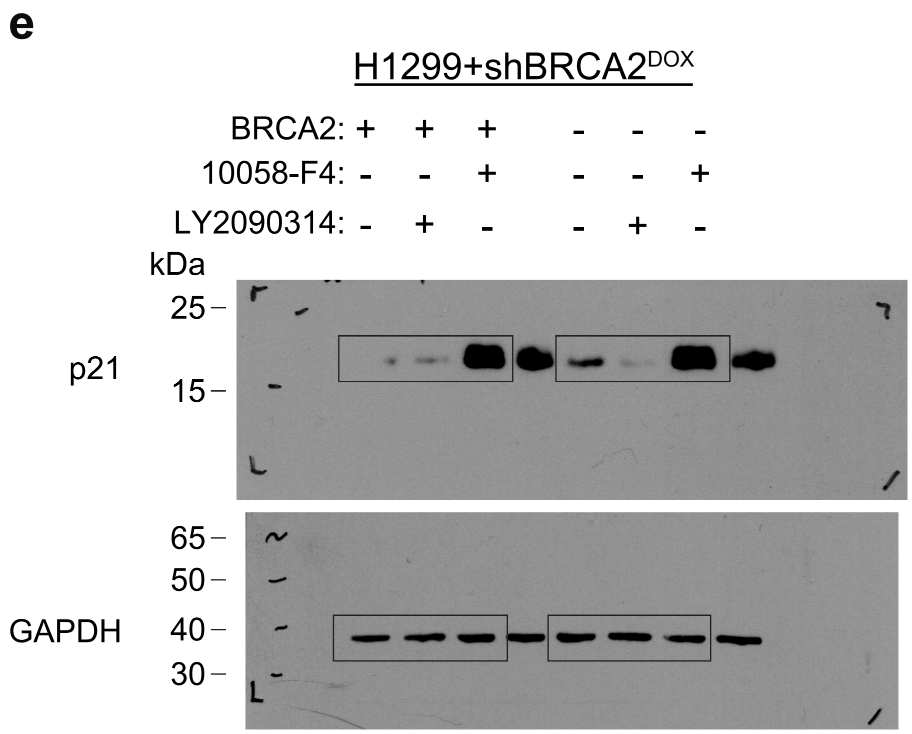

a

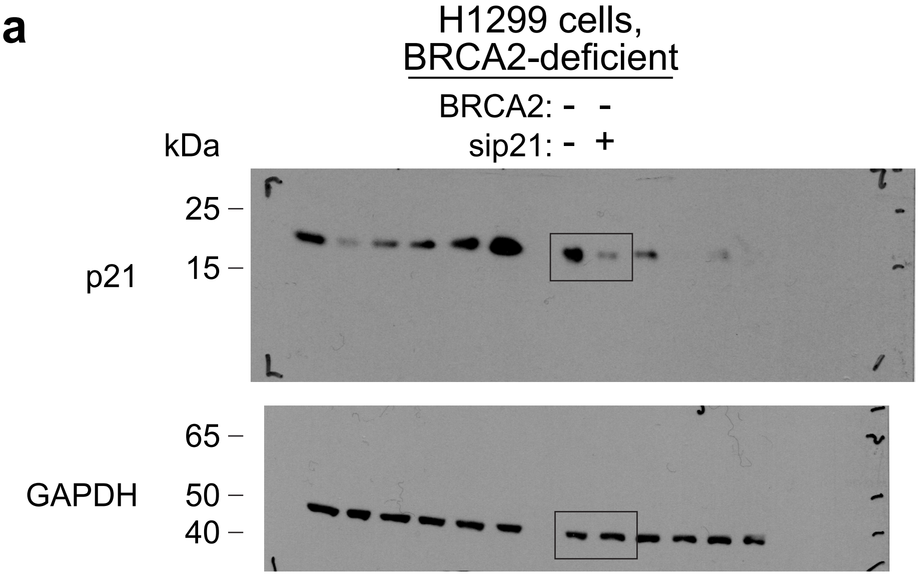
H1299 cells,
BRCA2-deficient
BRCA2: - -
sip21: - +
kDa
25
15
p21
65
50
40
GAPDH
